# Serum metabolite and metal ions profiles for breast cancer screening

**DOI:** 10.1038/s41598-024-73097-1

**Published:** 2024-10-19

**Authors:** Wojciech Wojtowicz, R. Tarkowski, A. Olczak, A. Szymczycha-Madeja, P. Pohl, A. Maciejczyk, Ł. Trembecki, R. Matkowski, Piotr Młynarz

**Affiliations:** 1 Department Biochemistry, Molecular Biology and Biotechnology, Faculty of Chemistry, Wroclaw University of Science and Technology, Wybrzeże Wyspiańskiego 27, 50-370 Wrocław, Poland; 2 Lower Silesian Oncology, Pulmonology and Hematology Center, Wroclaw, Poland; 3 Faculty of Electrical Engineering, Automatic Control and Informatics, Opole University of Technology, Opole, Poland; 4 Wroclaw Medical University, Wroclaw, Poland; 5 Department of Analytical Chemistry and Chemical Metallurgy, Wroclaw University of Science and Technology, Wroclaw, Poland

**Keywords:** Metabolomics, Biomarkers, Diseases, Oncology, Breast cancer, Cancer screening, NMR spectroscopy, Solution-state NMR, Mass spectrometry

## Abstract

Enhancing early-stage breast cancer detection requires integrating additional screening methods with current diagnostic imaging. Omics screening, using easily collectible serum samples, could serve as an initial step. Alongside biomarker identification capabilities, omics analysis allows for a comprehensive analysis of prevalent histological types—DCIS and IDC. Employing metabolomics, metallomics, and machine learning, could yield accurate screening models with valuable insights into organism responses. Serum samples of confirmed breast cancer patients were utilized to analyze metabolite and metal ion profiles, using two distinct analysis methods, proton NMR for metabolomics and ICP-OES for metallomics. The resulting responses were then subjected to discriminant analysis, progression biomarker exploration, examination of correlations between patients’ metabolites and metal ions, and the impact of age and menopause status. Measured NMR spectra and metabolite relative integrals were used to achieve statistically significant discrimination through MVA between breast cancer and control groups. The analysis identified 24 metabolites and 4 metal ions crucial for discrimination. Furthermore, four metabolites were associated with disease progression. Additionally, there were important correlations and relationships between metabolite relative integrals, metal ion concentrations, and age/menopausal status subgroups. Quantified relative integrals allowed for discrimination between studied subgroups, validated with a holdout set. Feature importance and statistical analysis for metabolomics and metallomics extracted a set of common entities which in combination provides valuable insights into ongoing molecular disturbances and disease progression.

**Supplementary Information:**

The online version contains supplementary material available at 10.1038/s41598-024-73097-1.

## Introduction

Breast cancer occurrence is increasing worldwide. Advances in medicine, improved diagnostics, screening tests, and heightened oncological vigilance in the society have significantly reduced mortality in developed countries. However, breast cancer still ranks first in the annual number of new cases in the USA and Poland (30% and 22.2% of all cases, respectively) and in most highly developed countries^1,2^. In the male population, there are significantly fewer cases, with about 1% of new breast cancer cases occurring among men^2^. Despite its high incidence breast cancer, is currently the second leading cause of cancer-related deaths, accounting for an estimated 15% in the US and 14.1% in Poland^1,2^. Breast cancer is a disease of civilization, with risk factors related to age, the presence of specific genes (including BRCA1, BRCA2, PALB2, CHEK2), family history of breast cancer, and long-term use of hormone therapies (e.g. hormone replacement therapy with estrogen)^3^. Routine screening is the most powerful tool for detecting the earliest stages of cancer. Early diagnosis allows for application of less debilitating and burdensome treatment methods. Detection of stage I and II breast cancer gives female patients over a 90% 5-year survival rate^4,5^. Currently used screening methods includeimaging techniques, histopathological analysis and cytological analysis, all of which are crucial for making a final diagnosis to be made^6,7^. These imaging methods are based on scientific evidence but require specific medical equipment operated by highly specialized and trained medical personnel. Additionally, they often requirepatients to travel to medical facilities. The idea of a readily available screening method based on serum samples with central verification sites could theoretically increase the ability to detect lesions at the earliest stages. Metabolomics is now a well-developed field of science that is increasingly used in medicine^8,9^. The development of its use in diagnostics may be the first step toward a quick, reliable method of identifying changes, allowing for the identification of individuals who require further diagnosis thus potentially serving as a screening method^8,9^. Breast cancer research on the variability of small molecule compounds has been conducted since 1993^10^. However, research focused mainly on verifying interchangeability in tissue samples until 2010, when the first NMR serum analyses were published^11^. Since first publication, significant progress has been made in the field of NMR metabolomics research on breast cancer, with a substantial number of valuable studies being published^12–14^. These studies have defined measurable metabolite profiles for breast cancer patient samples, facilitating the investigation of altered metabolites. The insights gained from this research have proven valuable in identifying tumor growth markers, detecting pre-diagnosis changes, distinguishing early-stage breast cancer (EBC) from metastatic breast cancer (MBC), differentiating subtypes, and assessing prognosis or risk of relapse, as well as evaluating the impact of treatment^11,15–37^.

This study utilized data science capabilities along with serum metabolomics and metallomics data to gain insight into the biochemistry of breast cancer and to explain biological variance. Clinical data such as age and menopausal status along with their correlations with metabolites and metal ions, were analyzed to provide detailed information about possible interactions occurring in the organism. Moreover, the generated information was assessed to verify the possibility of distinguishing between control and breast cancer individuals in the studied cohort.

## Materials and methods

### Sample collection

Peripheral venous blood samples were collected from all participants following an overnight fast. Blood samples were collected using serum tubes of the Sarstedt SMonovette system (Sarstedt AG & Co., Germany) and then centrifuged at 1000 × RCF for 15 min at 4 °C. Serum samples were stored in 1.5 mL Eppendorf safe-lock tubes and kept at − 80 °C until preparation.

The metabolomics study group included 161 patients with an established diagnosis of breast cancer and 161 samples from a control population of women, tested and without cancer diagnosis. The metal ions analysis study enrolled 164 breast cancer patients and 110 control samples. Clinical and demographic information for subjects qualified for metabolomics analysis is presented in Supplementary Tables S1–S3 and for metal ions analysis in Supplementary Tables S4 and S5. In both ‘omics analysis subjects were female patients of the Lower Silesian Oncology, Pulmonology and Hematology Center and serum samples were collected prior to treatment.

### Sample preparation for NMR measurements

The collected serum samples were thawed at room temperature and vortexed. Each serum sample (200 µL) was mixed with 400 µL saline solution (0.9% NaCl, w/v) containing 20% D_2_O (ARMAR AG, Döttingen, Swisserland). The mixture of serum and saline solution was vortexed and then centrifuged (10 min, 13840 × RCF, 4 °C). The supernatant (550 µL) from each sample was transferred into a 5 mm NMR tube (SP, 5 mm, ARMAR Chemicals, Leipzig, Germany). Samples were kept at 4 °C before 1 H NMR spectra acquisition.

### NMR measurements

The NMR spectra of serum were recorded at 300 K using an Avance II spectrometer (Bruker, GmBH, Germany) operating at proton frequency of 600.58 MHz. The 1D ^1^H NMR spectra were recorded using the CPMG pulse sequence with water presaturation (*cpmg1dpr*, *Bruker* notation). Pulse sequence parameters were set as follows: 128 transient scans, spin-echo delay of 400 µs; 80 loops; relaxation delay of 3.5 s; acquisition time of 2.73 s; time domain of 64k and spectral width of 20.01 ppm.

Two-dimensional NMR experiments (2D NMR) were recorded and processed for selected samples. Experiments performed included ^1^H−^1^H correlation spectroscopy (COSY), total correlation spectroscopy (TOCSY), and ^1^−^13^ C heteronuclear single quantum correlation (HSQC).

#### Metabolites identification

The metabolite resonance signals were identified in accordance with assignments published in the literature, availability in the Chenomx software (v 8.2 Chenomx Inc. Edmonton, Alberta, Canada) and on-line databases (Biological Magnetic Resonance Data Bank^38^ (www.bmrb.wisc.edu*)* and Human Metabolome Data Base^39^ (www.hmdb.ca). Correlation analysis between quantified resonance signals is presented in Supplementary Figs. S1–S3.

### Processing of NMR spectra for data analysis

The 1D ^1^H NMR spectra were processed with line broadening of 0.3 Hz, manually phased, and baseline corrected using MestReNova software (Mestrelab Research v 11.0). The spectra were and referenced to the glucose anomeric carbon signal group at δ = 5.225 ppm. The spectral range containing the water resonance signal was removed from the data matrix. The alignment of resonance signals was done using correlation optimized warping (COW)^40^ and icoshift algorithms^41^ implemented in Matlab (v. R2014a, Mathworks Inc., Natick, Massachusetts, USA). All spectra were normalized by Probabilistic Quotient Normalization (PQN)^42^. The relative integral of NMR measured metabolites was obtained as the sum of data points of the non-overlapping resonances or a cluster of partly overlapping resonances. The sample pool was split into training and testing sets using the Kennard-Stone algorithm with a 75–25% ratio, ensuring that both the, were training and testing sets, had balanced (1:1) ratio of samples from control and disease groups. The order of observations in the data matrix was randomized. All preparation of NMR measurements for further data analysis was carried out in Matlab software (Matlab v. R2014a, Mathworks, Inc., Natick, Massachusetts, USA).

### Metabolites datasets for univariate statistics

The control group (HC) vs. breast cancer (BC) statistics were calculated on a representative dataset containing 161 samples for each group, selected by the Kennard-Stone algorithm. The HC vs. infiltrating ductal carcinoma (IDC) vs. ductal carcinoma in situ (DCIS) statistics were calculated on a representative dataset containing 161 HC, 141 IDC, and 20 DCIS samples. The HC vs. Luminal A vs. Luminal B statistics were calculated on a representative data set containing 40 observations for each group, selected by the Kennard-Stone algorithm. The HC vs. Stage I vs. Stage II statistics were calculated on a representative dataset containing 161 samples for the HC group, 70 samples for Stage I and 59 samples for Stage II.

### Metabolomics univariate data analysis

The relative integrals (non-scaled) of assigned metabolites were used for calculations for univariate statistics. The Shapiro–Wilk test was calculated for normality verification for each variable. Potential outliers were identified was based on adjusted boxplots for non-normally distributed data or with the Generalized Extreme Studentized Deviate (ESD) test for normally distributed data (α = 0.05, 5 outliers), alongwith a visual inspection. Only extreme cases were marked as outliers. All statistical tests were calculated at a significance level of α = 0.05. Spearman’s Rho was used to verify possible correlations among variables. The ^1^H NMR relative integrals of metabolites were used to find age, menopause status and metabolites-metal ions correlations with the Spearman or Kendall correlation coefficient. Central tendency representations were based on the type of statistical test used, average group values were represented if the test was parametric or median if non-parametric. Changes between the relative integrals of metabolites of studied groups were verified by average percentage difference (APD) and median percentage difference (MPD).

### Two groups comparisons

The equality of variances for normally distributed data was tested using the Ftest. Depending on the results of normality and variance testing, either a parametric (equal/unequal variance Student’s t-test) or a nonparametric (Mann–Whitney–Wilcoxon test) test was performed. The False Discovery Rate (FDR) based on the Benjamini–Hochberg procedure was applied to the tested metabolites and metal ions. The classical univariate ROC curve analyses were prepared using the Matlab statistical toolbox (Matlab v. R2014a, MathWorks, Inc., Natick, Massachusetts, USA) perfcurve function for the selected metabolites.

### Multiple groups’ comparisons

Homogeneity of variation was tested using Levene’s test. Depending on the results of normality and variance tests, either a parametric ANOVA test with Tukey’s HSD correction or a non-parametric Kruskal–Wallis test with Dunn–Šidák correction was used to verify statistically significant differences between the studied groups.

### Metabolomics multivariate data analysis

The MVA analysis was carried out on two types of data: metabolites’ relative integral data, with or without unknown resonance signals. In both types of data, the same procedure of data preparation was implemented.

All the relative integral variables were scaled to unit variance (UV). The Principal Component Analysis (PCA), The Partial Least Squares regression (PLSR) and Orthogonal Projections to Latent Structures Discriminant Analysis (OPLS-DA) calculations were performed using SIMCA 17.0.0.2 software (Sartorius Stedim Biotech, 2021, Goettingen, Germany). PCA was applied for data overview and outlier detection. The MVA data visualization included an ellipse marking Hotelling’s T2 range (95%).

The Orthogonal Projections to Latent Structures Discriminant Analysis with a 7-fold cross validation procedure was used to determine differences between the studied groups. The number of latent variables was selected based on cross-validated improvement of Q^2^ (cum) < 0.01. The reliability of the OPLSDA models was assessed by analysis of variance of cross-​validated residuals (CVANOVA) at a significance level of α = 0.05. The VIP values for OPLS-DA models were calculated based on a 7-fold cross-validation procedure (VIPcv-7). Specificity and sensitivity were determined according to sample class prediction using the 7-fold cross-validated predicted values from the fitted Ypredcv and YpredPScv (implemented in SIMCA 17.0.0.2 software) for training and testing observations in the model.

Partial Least Squares regression was used with a 7fold cross-validation procedure for relative integral data and subjects’ age as the Y-block. The importance of variables in PLSR was calculated using VIP values. The number of latent variables was determined by the lowest RMSECV.

The XGBoost (XGB) and Artificial Neural Network (ANN) models were trained for 50 iterations with a random seed, and the average performance was reported. Feature importance was reported as an average score across the trained model iterations.

The XGBoost (v. 1.7.5) model was trained on a binary logistic objective with a focus on AUC as the evaluation metric. The training procedure included early stopping after 50 rounds and a total of 150 boosting rounds. Interpretation of important variables in the XGB model was carried out using the built-in function option for variables’ total gain.

The Artificial Neural Network was built using the Keras library (v. 2.12.0), within the TensorFlow (v 2.12.3) machine learning framework in the Python (v. 3.10.7) programming language. A sequential data model was initialized with four layers. The first layer was an initial layer, with artificial neurons equal to the number of x variables and matching the number of input values. The activation function for the first layer was set to Softmax. Generated tensors were based on the uniform distribution initializer with minval set to 0 and maxval set to 1. The first layer was followed by two hidden layers, the first with 40 neurons and the Softmax activation function, and the second with 20 neurons and the Rectified Linear Unit (ReLU) function. The output layer used the sigmoid activation function returning a single output as a fractional value between zero and one. The model was compiled with the binary corssentropy loss function, and the Adam optimizer. The main metric followed in the training process was accuracy. ANNs were trained with an epoch size set to 500 utilizing three callback functions: model checkpoint monitoring validation set accuracy, learning rate reduction on loss value, and early stopping based on validation loss. The models’ important variables were interpreted using Shapley Additive Explanations with KernelExplainer on default settings (SHAP, v. 0.41.0).

The prediction performance of the trained models was assessed with receiver operating characteristic (ROC) curves and area under the curve (AUC) values utilized scikit-learn (v. 1.2.2) and visualized with matplotlib (v 3.5.3).

### ICP-OES measurements

An Agilent bench-top optical emission spectrometer of an axially viewed Ar-ICP, model 720, was used to determine the concentrations of selected elements, i.e. As, Ca, Cd, Co, Cr, Cu, Fe, Mg, Ni, Pb, Se and Zn. The instrument was equipped with a 4-channel peristaltic pump, a high-resolution echelle-type polychromator and a VistaChip II CCD detector. A standard, one-piece, low-flow, extended quartz torch with an injector tube (2.4 mm ID) was used to sustain and operate the plasma. The torch was combined with a single-pass glass cyclonic spray chamber. A high-tech engineering polymer (PFA and PEEK) OneNeb concentric nebulizer was mounted into the spray chamber and applied to introduce all the solutions by pneumatic nebulization. The following operating instrument settings were applied: RF power of 1.2 kW, the gas flow rates of 15.0 (plasma), 1.5 (auxiliary) and 0.75 L min^− 1^ (nebulizer), the sample flow rate of 0.75 mL min^− 1^, stabilization and sample uptake delays of 15 and 30 s, respectively, the rinse and replicate times of 10 and 1 s (3 replicates), respectively. The most prominent and free from vast spectral interferences analytical lines were selected: As 193.7, Ca 317.9, Cd 226.5, Co 238.9, Cr 267.7, Cu 327.4, Fe 259.9, Mg 285.2, Ni 231.6, Pb 220.3, Se 196.0 and Zn 213.8 nm. The background corrected intensities of lines (means of three replicates) were used for the calibration and quantification of the concentrations of As, Ca, Cd, Co, Cr, Cu, Fe, Mg, Ni, Pb, Se, and Zn. The working standard solutions for the five-point calibration were within the range of 0.1–5.0 µg mL^− 1^.

### Sample preparation for ICP-OES measurements

A Merck Certipur^®^ multi-elemental stock (1000 mg L^−1^) ICP standard solution XVI was used to prepare the matrix-matched standard solutions for the calibration of ICP OES. De-ionized water from an EASYpure™ water purification system (Barnstead Corp., USA) was used to prepare all the solutions (standards and samples). Prior to the spectrometric measurements, the analyzed serum samples were × 10 times diluted with de-ionized water and analyzed against the matrix-matched standard solutions (in reference to the content of K and Na ions in the serum samples). To assess the reliability of the sample preparation procedure and the calibration strategy used, a recovery study was conducted and quantitative recoveries were obtained.

### ICP-OES data set preparation

The concentrations of detected serum metal ions were connected to the corresponding samples form the metabolomics analysis. Variables below the limit of detection (LOD) were substituted with half of detection limit value (LOD As < 0.005, Se < 0.005). The main analyses were conducted on data from 273 observations, 109 from the control group and 164 from the breast cancer group. For the combined metabolomicsmetallomics analysis, a total of 236 samples matched the metabolomics dataset, with the breast cancer group containing154 samples and the control group containing 82 samples.

### Metal ions concentrations data analysis

The concentrations of metal ions along with matching samples’ ^1^H NMR relative integral (R.I.) of metabolites, were used to find age, menopause status, and metabolite-metal ions correlations. The correlation coefficient was calculated using Spearman’s method for non-censored data and Kendall’s tau-b for left-side censored data. Comparisons between two groups for censored data were carried out using two-sided Mann–Whitney–Wilcoxon test. The central tendency for censored data was calculated based on the percentage of censored data, Kaplan–Meier (K–M) for less than 50% and Maximum Likelihood Estimation (MLE) for 50–80%. Calculations and graphical representations were prepared using the Matlab Statistical Toolbox (Matlab v.R2019a, MathWorks, Inc., City, Country). In the conducted analysis, metal ions, with more than 60% of observations below the limit of detection (LOD), were removed from the analysis (Supplementary Table S6).

### Metabolite set enrichment analysis

Biochemical pathway analysis was carried out with use of MetaboAnalyst 5.0^43^. The dataset contains 318 observations, and the metabolites’ relative integrals were a priori UV scaled for MSEA. Selected Quantitative Enrichment Analysis (QEA) was calculated without lipid regions and covered only identified metabolite concentration regions with single metabolite IDs. The metabolite set library was set on SMPDB. The enrichment analysis was performed using the package globaltest.

## Results

The signal composition on the ^1^H NMR of the serum sample allowed for the quantification of 31 identified metabolites: leucine, valine, isoleucine, 3-hydroxybutyrate, lactate, alanine, lysine, acetate, NAC, glutamine, acetone, lipids, acetoacetate, glutamate, pyruvate, citrate, dimethylamine, creatine, creatinine, choline, O-phosphocholine, GPC, glucose, betaine, glycine, glycerol, tyrosine, histidine, phenylalanine, formate, four unknown resonance signals and eight different spectral ranges assigned to various types of lipids (Supplementary Table S7).

### Comparison of breast cancer and control group

The metabolites’ relative integral data were divided into two dataset types. The first dataset contained identified metabolites along with unknown resonance signals (R.I.) and the second dataset contained variables with confirmed level two identification (R.I. w/o unknowns). These different data sources were then used to train separate discrimination models. The main dataset of 322 samples was split into two parts based on the Kennard–Stone algorithm^44^, where the training set contained equal-sized groups of 121 control samples (HC) and 121 breast cancer samples (BC) (overall, 242 training samples) and the testing set included 40 HC and 40 BC (80 testing samples). Durning the process of multivariate data analysis based on exploratory analysis, four samples were removed as significant outlying observations from the training set, two from the healthy control group and two from the breast cancer group, leading to data calculations based on an overall 318 samples. These sample sets were used in all training and testing models. For breast cancer screening purposes three different models (OPLS-DA, ANN, and XGB) were trained to assess predictive performance along with the juxtaposition of the identified important relative integrals of the resonance signals.

The calculated OPLS-DA models obtained Q^2^(cum) values above 0.5, partially confirming the good discrimination quality between the control group and breast cancer (Table 1, Supplementary Table S1). The reliability of the OPLS-DA models was tested with CV-ANOVA, and the models passed validation (Table 1).

**Table 1:** The multivariate data analysis models’ summary includes the models’ parameters, the values of the CV-ANOVA and the AUC values for individual datasets.

Data type	Model type	LV	*N*=	*R*^2^X(cum)	*R*^2^Y(cum)	Q^2^(cum)	CV-ANOVA
R.I.	OPLS-DA	1 + 3 + 0	238	0.477	0.657	0.552	5.17E−35
R.I. w/o unknowns	OPLS-DA	1 + 3 + 0	238	0.505	0.649	0.549	1.12E–34

*R.I.* relative integral.

**Fig. 1:**
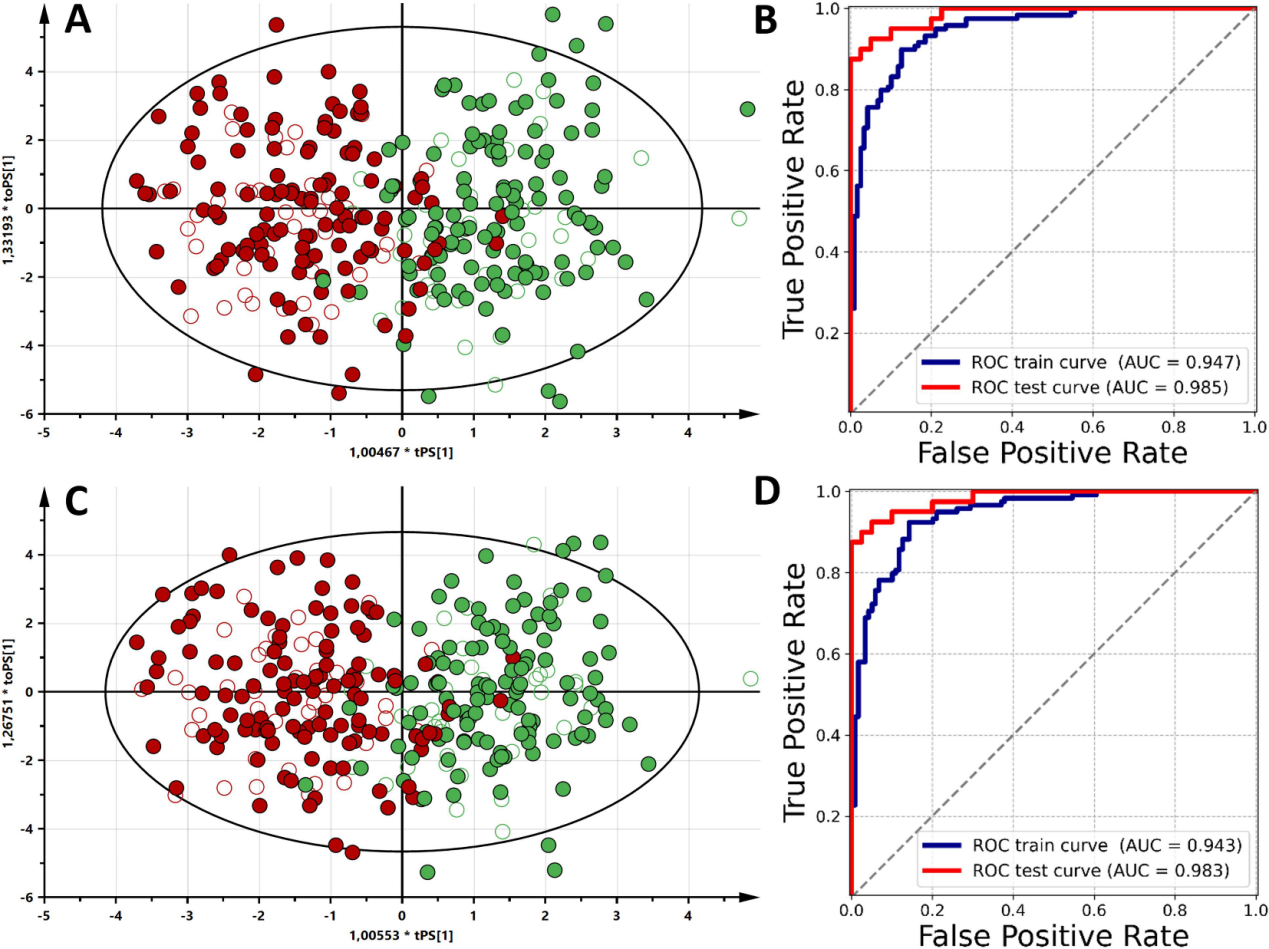
The OPLS-DA models for all data types used in the study, along with ROC curves for predictive potential. (**A**,**B**) Relative integral of metabolites, (**C**,**D**) Relative integral of metabolites without unknown resonance signals.

The representation of OPLS-DA model scores for both data sources, along with corresponding ROC plots for the training and testing sets is shown in Fig. 1.

For the ANNs and XGBoost, the applied training settings were determined to maximize the accuracy of predictions. The training of ANN and XGBoost models proceeded with the original training data (*N* = 238) obtained by the Kennard-Stone (K-S) algorithm, with an additional split into training and validation subsets, sequentially 85% and 15%.

Both data sources (R.I. and R.I. w/o unknowns) led to quality models with high predictive potential verified by the testing sample set and represented by AUC ROC values. For the calculated OPLS-DA models, AUC ROC reached above 0.943 in the training set and over 0.983 in the testing set (Fig. 1; Table 2). Further quality assessment for different training data types showed that the OPLS-DA models on R.I. data with unknown resonance signals displayed the best predictive performance (AUC_R.I.test_ = 0.985, AUC_R.I.w/o Unknowns test_ = 0.983) (Table 1). The ANNs on R.I. data obtained the mean AUC_train_ = 0.962, mean AUC_test_ = 0.958 (Table 2) and on data R.I. without unknown resonance signals data mean AUC_train_ = 0.977 and mean AUC_test_ = 0.971 (Table 2). For XGB, the model on R.I. data obtained mean AUC_train_ = 0.994, median AUC_test_ = 0.979 (Table 2) and when trained on R.I. without unknown resonance signals, the data model reached mean AUC_train_ = 0.994 and mean AUC_test_ = 0.975 (Table 2).

**Table 2:** The models’ AUC ROC and accuracy summary in relation to training and testing set samples. Classification threshold for accuracy < 0.5.

Model type	OPLS-DA		ANN		XGB	
Dataset (training set, *N* = 238/ test set, *N* = 80)	*R*.I.	*R*.I. w/o Unknowns	*R*.I.	*R*.I. w/o Unknowns	*R*.I.	*R*.I. w/o Unknowns
Mean AUC train set (%)	0.947	0.943	0.962	0.977	0.994	0.994
Mean AUC test set (%)	0.985	0.983	0.958	0.971	0.979	0.975
Mean accuracy train set (%)	0.882	0.874	0.929	0.950	0.971	0.970
Mean accuracy test set (%)	0.925	0.938	0.888	0.898	0.907	0.903

*R.I.* Relative integrals, *AUC* Area under curve.

The ROC AUC analysis displayed overall high predictive performance for each trained model. Among the selected model types, the highest predictive potential based on AUCtrain and AUCtest was observed in the XGB model trained on data with unknown resonance signals (Table 2). However, the highest AUCtest was observed in the cross-validated OPLS-DA. A summary of prediction accuracy and comparisons of AUCs of trained models is represented in Table 2.

**Fig. 2:**
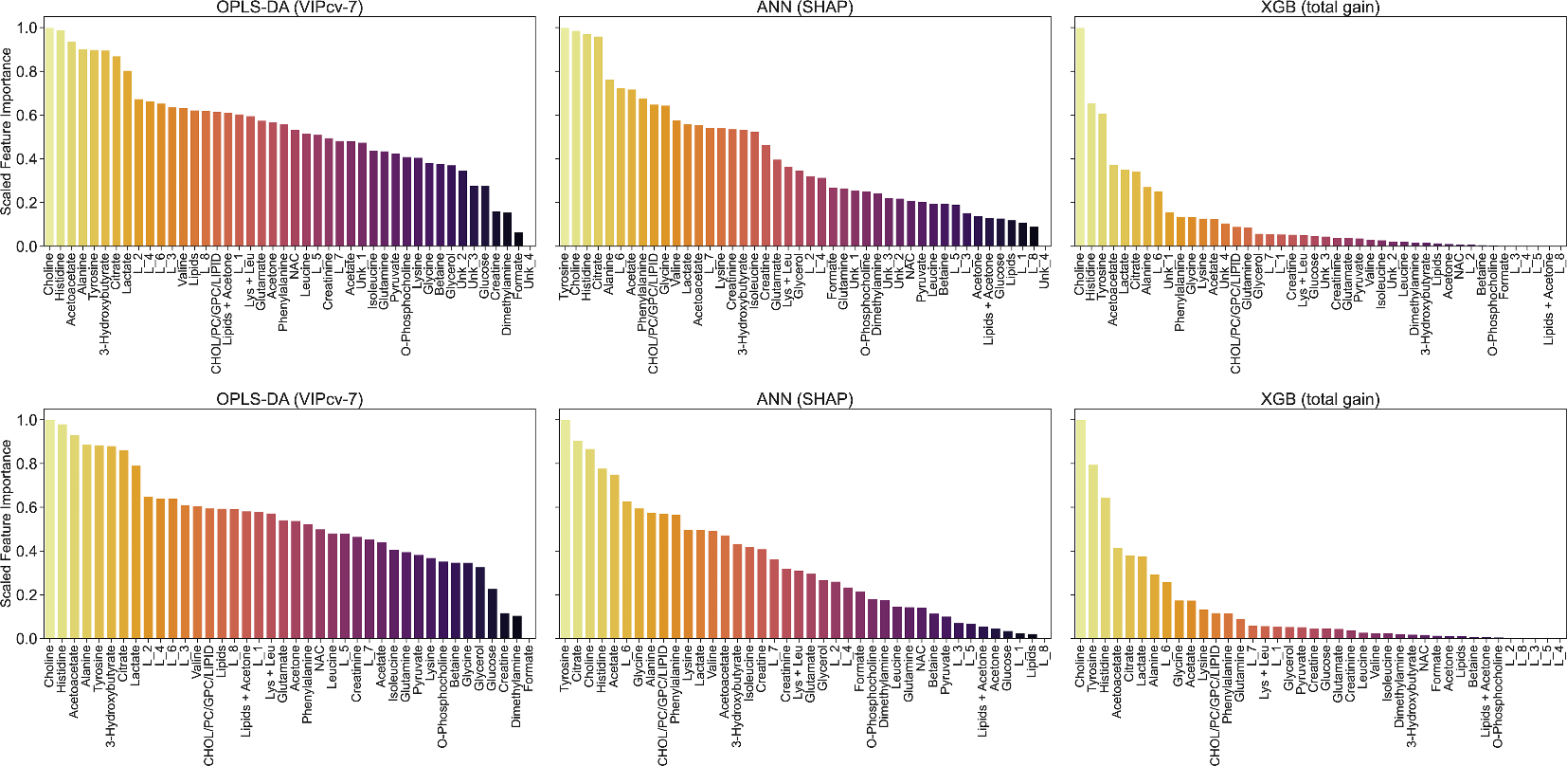
Scaled feature importance scores in dependence of model and data type. Top row dataset with unknown resonce singals (R.I.), bottom row, dataset without unknown resonance singals (R.I. w/o Unknowns).

The trained models were investigated to select the most influential features in each model architecture. For selecting important features, different models’ interpretability and explainability methods were implemented. For OPLS-DA, the 7-fold cross-validated VIP scores were used, mean SHAP values were calculated for ANN model iterations and mean total feature gain was used for XGB iterations. The highest impact features are presented in Fig. 2 in descending order. The complete list of variables and their corresponding importance values are presented in Supplementary Table S8.

### Univariate analysis for relative integral of metabolites

#### Comparison of breast cancer and control group

The univariate analysis focused on identifying a set of main biochemical compounds responsible for differentiation between control groups (HC) and breast cancer (BC). Out of the whole set of quantified relative integral metabolites, 24 of 44 had statistically significant differences in relative integrals between the two main groups after the FDR procedure (Table 3). Of the statistically significant differences in metabolites’ relative integrals, eight were elevated in the breast cancer group and 16 were decreased compared to the control group.

**Table 3:** Summary of statistically important in univariate statistics for metabolites relative integrals for HC vs. BC comparison, sorted by FDR adjusted p value (< 0.05).

No.	Metabolite	Central tendency		Relative standard deviation (%)		Average percentage difference (%)	*p* value	FDR adjusted *p* value
		HC	BC	HC	BC	HC vs. BC	HC vs. BC	HC vs. BC
1	Choline^a^	0.153	0.175	13.008	13.017	13.34	5.68E–18	1.25E−16
2	Histidine^b^	0.043	0.037	13.444	17.205	− 15.32	5.61E–18	1.25E−16
3	Tyrosine^b^	0.094	0.082	15.490	15.588	− 15.24	8.59E–17	1.26E−15
4	Acetoacetate^b^	0.100	0.124	16.054	37.622	30.54	1.99E–14	2.18E−13
5	Alanine^b^	0.856	0.711	15.538	16.714	− 14.70	5.50E–14	4.84E−13
6	3-Hydroxybutyrate^b^	0.579	0.671	15.301	24.202	18.21	3.65E–13	2.68E−12
7	Citrate^b^	0.068	0.078	19.550	20.423	14.94	3.00E–11	1.89E−10
8	Lactate^b^	4.465	3.691	21.461	25.200	− 15.31	9.22E–10	5.07E−09
9	L_6^a^	3.621	3.276	18.979	20.497	− 10.00	7.51E−06	3.67E−05
10	Acetate^b^	0.076	0.071	22.945	20.335	− 10.38	1.38E−05	6.06E−05
11	Glutamate^b^	0.132	0.124	15.564	15.171	− 7.44	1.70E−05	6.80E−05
12	Glycine^b^	0.412	0.372	24.959	25.647	− 11.57	7.79E−05	2.86E−04
13	Creatinine^b^	0.179	0.168	14.140	12.690	− 5.93	1.32E−04	3.97E−04
14	CHOL/PC/GPC/LIPID^a^	4.351	4.047	16.412	17.425	− 7.25	1.44E−04	3.97E−04
15	L_7 (glycerol of lipids)^b^	0.036	0.040	25.499	21.495	9.44	1.23E−04	3.97E−04
16	Phenylalanine^a^	0.091	0.087	10.812	14.481	− 5.45	1.42E−04	3.97E−04
17	Valine^b^	0.502	0.474	12.964	12.613	− 5.34	2.45E−04	6.35E−04
18	Unk_2^b^	0.090	0.093	17.778	15.210	4.91	1.38E−03	3.20E−03
19	Lys + Leu^a^	0.690	0.669	8.894	8.314	− 3.11	1.35E−03	3.20E−03
20	Unk_4^b^	0.089	0.080	26.886	33.705	− 7.90	2.76E−03	6.07E−03
21	Acetone^b^	0.205	0.229	35.381	34.057	9.54	5.26E−03	1.10E−02
22	Glycerol^b^	0.085	0.088	16.948	16.079	4.08	1.38E−02	2.77E−02
23	Unk_3 (Singlet)^b^	0.045	0.040	31.073	32.893	− 4.75	1.65E−02	3.16E−02
24	Pyruvate^b^	0.091	0.085	28.833	28.716	− 7.46	1.81E−02	3.32E−02

^a^Parametric test, ^b^non-parametric test, Central Tendency- mean for parametric, median for non-parametric.

**Fig. 3:**
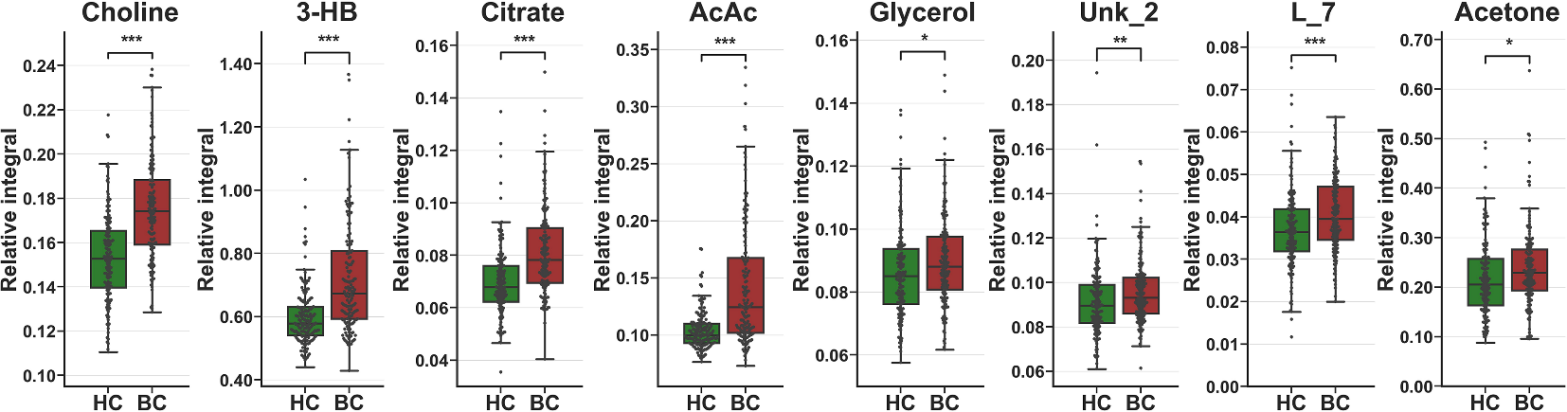
Boxplot with data points for statistically important metabolites elevated in the BC group (HC vs. BC comparison). Whiskers—1.5 × IQR; bar—average; box—Q1–Q3 interquartile range. p value annotation, *<0.05, **<0.01, ***< 0.001. Detailed values are in Table 3.

The elevated relative integrals of small molecular compounds in the breast cancer group were observed for choline, acetoacetate, 3hydroxybutyrate, citrate, L_7, Unk_2, acetone, and glycerol (Table 3; Fig. 3).

**Fig. 4:**
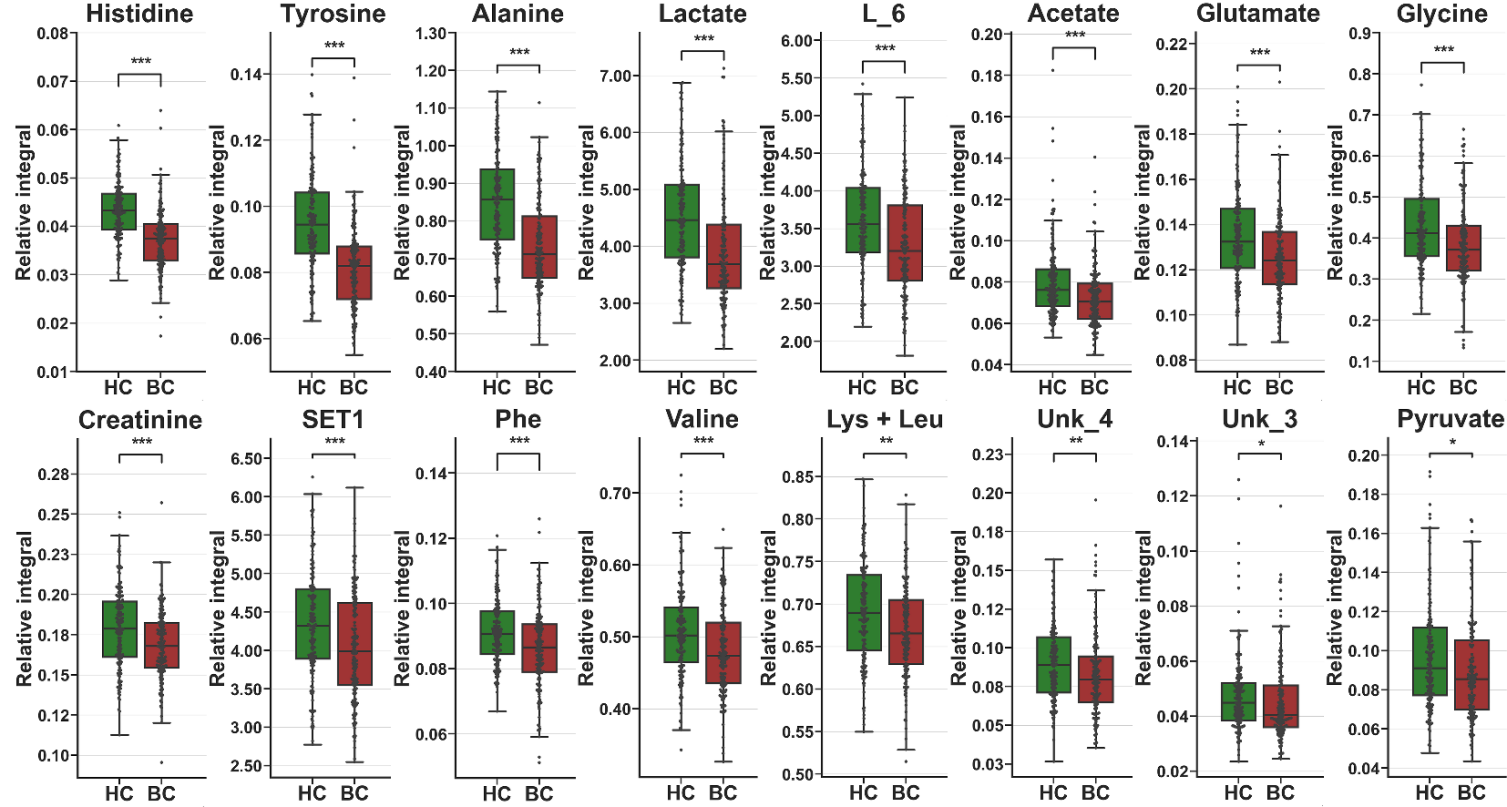
Boxplot with data points for statistically important metabolites that were decreased in the BC group (HC vs. BC comparison). Whiskers—1.5 × IQR; bar—average; box—Q1–Q3 interquartile range. SET1—CHOL/PC/GPC/LIPID. ***< 0.001, **<0.01, *<0.05. Detailed values in Table 3.

The reduced relative integral values observed in the BC group were for histidine, tyrosine, alanine, lactate, L_6, acetate, glutamate, glycine, creatinine, CHOL/PC/GPC/LIPID, phenylalanine, valine, lysine + leucine, Unk_4, Unk_3, and pyruvate (Table 3; Fig. 4).

### Metal ions analysis

**Fig. 5:**
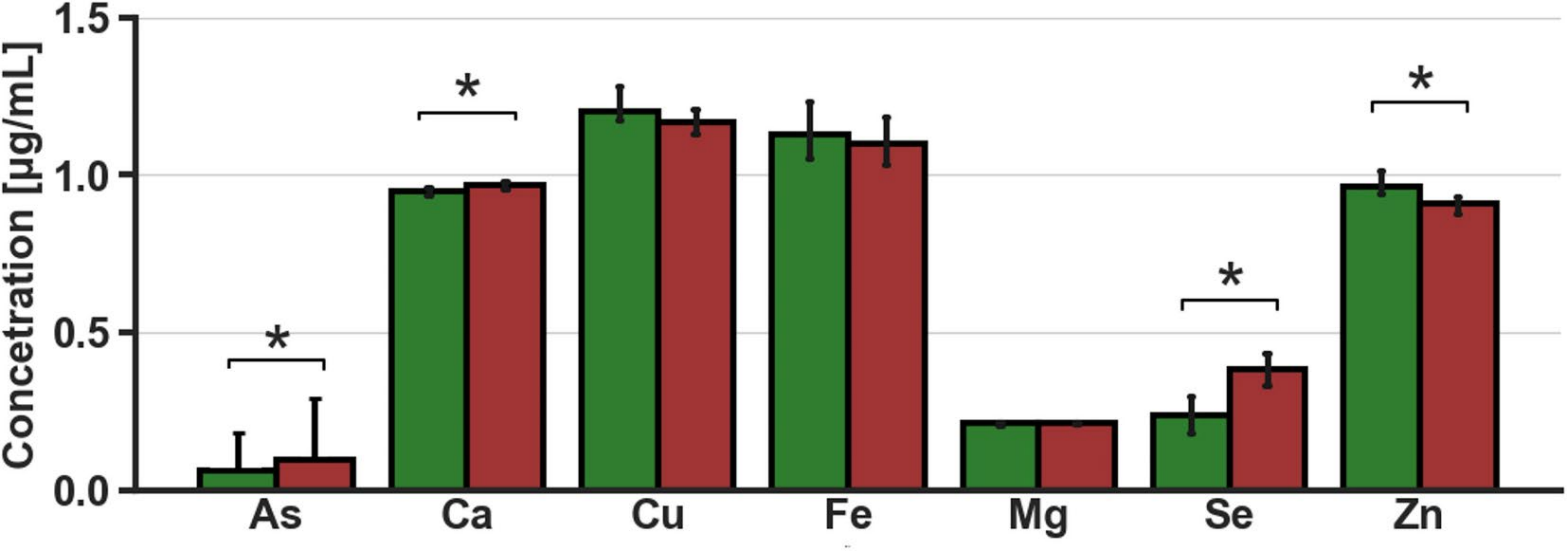
The median concentration of metal ions measured in serum samples for control vs. breast cancer; Ca and Mg concertations are presented in 10^− 2^ values and As 10^1^. Error bar—confidence interval 0.95 for Ca, Cu, Fe, Mg, and Zn and the quantile 0.75 from K–M estimation for As. Healthy control—green, breast cancer—red. *FDR adjusted p-value < 0.05.

The measured serum metal ion concentrations showed statistically significant differences between the studied primary groups (HC, BC). Four of the seven quantified metal ion concentrations had statistically significant diffrences—As, Ca, Se, and Zn. The breast cancer group exhibited elevated levels of As, Ca and Se, in while the concentration of Zn was reduced compared to the control group (Fig. 5).

Moreover, possible correlations between quantified metal ion concentrations and the relative integrals of small molecular compounds were verified by Spearman or Kendall correlation coefficients (Supplementary Fig. S4). The correlation analysis was conducted on three different groups: the control group, the breast cancer group and a combined dataset of control and breast cancer groups (Supplementary Fig. S4). The metal ion concentrations statistically significant in the HC vs. BC comparison (As, Ca, Se, and Zn) showed important correlations with relative integrals in five cases for the control group. Four positive correlations were observed between Ca and citrate (0.300), Ca and choline (0.251), Ca and creatinine (0.245) and Ca and lysine (0.225) and one negative correlation between Ca and L_1 (− 0.228) (LDL/VLDL, cluster at 0.85 ppm). In the breast cancer group, nine statistically significant correlations were obtained. Positive correlations were observed for Zn and valine (0.171), As and tyrosine (0.145), S and -acetone (0.141), Se and tyrosine (0.134). Negative correlations were found for Zn andbetaine (− 0.197), Ca andcreatine (− 0.164), As-glycine (− 0.127) and unk_4 (− 0.116). In the combined dataset, 18 correlations were statistically significant. Eleven of these were related to Zn concentration: histidine (0.263), alanine (0.253), Unk_4 (0.180), tyrosine (0.161), glucose (− 0.132), betaine (– 0.141), creatinine (– 0.155), valine (– 0.179), acetoacetate (– 0.2), Unk_2 (– 0.217), and 3-HB (− 0.253)) Four correlations were related to Se: relative integrals of acetone (0.094), creatine (– 0.091), alanine (– 0.115), and Unk_4 (– 0.119). For As, the correlations werewith glutamine (0.101) and glycine (– 0.126) and For Ca, the correlation was with citrate (0.180) relative integral. Nonetheless, comprehensive correlation analysis did not show any strong relationships (> 0.7 rho) among any studied groups within the important metal ions from the HC vs. BC discrimination (As, Ca, Se, and Zn) (Supplementary Fig. S4).

#### Metabolites related to prediction of disease progression

Although discrimination between HC and BC was possible, another important factor is metabolites associated with disease progression. To verify the first and most prominent discrimination, we performed a two-group statistical analysis of differences in the relative integrals of metabolites for stage I and II invasive breast cancer. In univariate analysis, four metabolites’ relative integral diffrences were statistically significant in differentiating between stage I and II: 3hydroxybutyrate, acetoacetate, glycerol, and lysine. Importantly, after FDR correction, none of the relative integrals had statistically significant diffrences in the stage I vs. II comparison.

The APD and MPD showed that three of the four metabolites had increased values through disease progression. Only lysine’s relative integral showed a different trend, with a decrease for stage II in compared to HC or stage I. The ROC curve for these metabolites showed that the best discrimination was with 3-hydroxybutyrate and acetoacetate, with both AUCs for each comparison (HC vs. Stage I, HC vs. Stage II, Stage I vs. Stage II) ranging from 0.620 (Acetoacetate, Stage I vs. Stage II) to 0.809 (Acetoacetate, HC vs. Stage II) (Table 4).

**Table 4:** Summary of average and median percentage difference of statistically important metabolites between invasive breast cancers stage I and stage II.

Metabolite	Comparison	APD [%]	MPD [%]	AUC
3-Hydroxybutyrate^b^	HC vs. Stage I	12.482*	9.476*	0.682
	HC vs. Stage II	24.108*	18.068*	0.795
	Stage I vs. Stage II	11.714*	8.629*	0.635
Acetoacetate^b^	HC vs. Stage I	35.878*	21.507*	0.692
	HC vs. Stage II	48.528*	35.358*	0.809
	Stage I vs. Stage II	13.226*	14.119*	0.620
Glycerol^b^	HC vs. Stage I	2.097	1.482	0.536
	HC vs. Stage II	6.947*	8.131*	0.639
	Stage I vs. Stage II	4.852*	6.652*	0.612
Lysine^a^	HC vs. Stage I	3.344	4.058	0.574
	HC vs. Stage II	− 1.523	1.543	0.528
	Stage I vs. Stage II	− 4.867*	− 2.515*	0.614

*Statistically important metabolites, ^a^parametric test, ^b^non-parametric test.

**Fig. 6:**
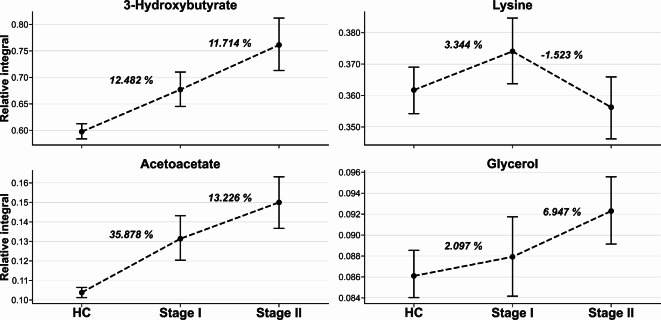
The point plot for selected metabolites with representation of average relative integral with 95% confidence interval. The values between groups refer to the average percentage difference between them.

The metabolites and their APD changes according to disease progression are shown in the point plot below. The difference in the relative integral of 3-hydroxybutyrate between HC groups and stage I is 12.482%, and between stage I and II is 11.714% (Fig. 6).

### Comparison of IDC, DCIS and control group

Comparisons with studied cancer subtypes of HC vs. DCIS vs. IDC, due to the large disparity (Supplementary Table S1) in sample sizes, nonparametric methods were used to verify potential differences.

The Kruskal–Wallis test showed a set of 23 metabolites that were statistically significant (Table 5). Specifically, in multiple group comparisons based on Dunn–Šidák correction, 13 of the 23 statistically significant metabolites in the HC vs. IDC comparison were precisely connected to the IDC group: glutamate, acetate, L_6, glycine, creatinine, L_7 (glycerol of lipids), valine, CHOL/PC/GPC/LIPID, Unk_2, Unk_4, lysine + leucine, acetone, and glycerol. For HC vs. DICS, 9 metabolites were statistically significant; however, all of them were also significant for HC vs. IDC: histidine, tyrosine, choline, acetoacetate, alanine, 3-hydroxybutyrate, citrate, lactate, and phenylalanine. Moreover, only one metabolite—creatine—was unique to the IDC vs. DCIS comparison.

**Table 5:** Summary of Kruskal–Wallis test for relative integrals between groups HC, IDC, and DCIS with Dunn–Šidák correction post hoc. Metabolites are sorted by p value.

No.	Metabolite	Kruskal–Wallis test	Median relative integral			Relative standard deviation (%)			Dunn–Šidák significance		
		*p* value	HC	IDC	DCIS	HC	IDC	DCIS	HC vs. IDC	HC vs. DCIS	IDC vs. DCIS
1	Histidine	4.44E−17	0.043	0.037	0.038	13.444	17.772	12.980	**+**	**+**	−
2	Tyrosine	6.78E−16	0.094	0.082	0.084	15.490	15.336	17.286	**+**	**+**	−
3	Choline	8.90E−16	0.153	0.173	0.179	13.008	13.149	12.174	**+**	**+**	−
4	Acetoacetate	1.93E−13	0.100	0.124	0.117	16.054	36.660	43.423	**+**	**+**	−
5	Alanine	4.74E−13	0.856	0.705	0.721	15.538	16.921	15.586	**+**	**+**	−
6	3−Hydroxybutyrate	3.37E−12	0.597	0.715	0.734	15.301	23.887	26.597	**+**	**+**	−
7	Citrate	2.20E−10	0.068	0.078	0.078	19.550	21.186	14.670	**+**	**+**	−
8	Lactate	6.03E−09	4.465	3.703	3.572	21.461	24.968	26.847	**+**	**+**	−
9	Glutamate	2.83E−05	0.132	0.123	0.127	15.564	15.274	14.131	**+**	−	−
10	Acetate	5.51E−05	0.076	0.070	0.075	22.945	21.175	14.103	**+**	−	−
11	L_6	7.12E−05	3.562	3.191	3.233	18.979	20.975	17.370	**+**	**−**	−
12	Glycine	3.85E−04	0.412	0.370	0.387	24.959	25.755	25.538	**+**	−	−
13	Creatinine	4.84E−04	0.179	0.167	0.175	14.140	13.127	9.398	**+**	−	−
14	L_7 (glycerol of lipids)	5.52E−04	0.036	0.040	0.038	25.499	21.822	18.714	**+**	−	−
15	Phenylalanine	6.37E−04	0.091	0.087	0.083	10.812	14.480	14.009	**+**	**+**	−
16	Valine	7.77E−04	0.502	0.471	0.475	12.964	12.815	11.235	**+**	−	−
17	CHOL/PC/GPC/LIPID	1.07E−03	4.326	3.967	4.063	16.412	17.879	14.387	**+**	−	−
18	Unk_2	4.50E−03	0.090	0.094	0.093	17.778	15.486	13.169	**+**	−	−
19	Unk_4	6.09E−03	0.089	0.078	0.084	26.886	34.114	31.170	**+**	−	−
20	Lys + Leu	8.54E−03	0.690	0.665	0.678	8.894	8.653	5.710	**+**	−	−
21	Acetone	2.03E−02	0.205	0.229	0.242	35.381	34.608	30.363	**+**	−	−
22	Creatine	4.06E−02	0.160	0.151	0.175	18.971	20.116	16.325	−	−	**+**
23	Glycerol	4.18E−02	0.085	0.088	0.086	16.948	16.314	14.460	**+**	−	−

+ Statistical difference between groups (< 0.05), − no statistical significance between groups (> 0.05).

### Comparison of DCIS, Luminal A, Luminal B and control group

The IDC group in the study containeda set of samples from luminal A (LA) and luminal B (LB). The criteria for qualifying tumors into LA and LB molecular subtypes are based on the expression of steroid receptors (estrogen and progesterone), HER2 and Ki-67 evaluated in the tumor tissue on histopathological examination. Both groups were used to highlight differences in metabolite relative integrals in IDC subtypes.

All metabolites were verified in terms of variance and distribution to fulfil assumptions for ANOVA or Kruskal–Wallis test. The differences in statistically significant metabolites were followed up by multiple comparison tests.

Overall, twelve small molecular compounds were statistically significant in multigroup comparisons of HC vs. Luminal A vs. Luminal B vs. DCIS (Table 6). Eight metabolites were highlighted as significant for both tested groups (HC vs. LA; HC vs. LB)—choline, acetoacetate, alanine, lactate, histidine, 3-hydroxybutyrate, tyrosine, and unk_1. Similarly, in HC vs. DCIS but with the exclusion of histidine and tyrosine. Three metabolites were not common for the verified subgroup comparisons—citrate for luminal B and DCIS groups and in the case of luminal A, glutamate and Ophosphocholine (Table 6). Only single metabolite creatine in multigroup comparisons was statistically significant between lesion groups in DCIS vs. LB (Table 6).

**Table 6:** Summary of Kruskal–Wallis or ANOVA tests for relative integral between groups HC, DCIS, Luminal A (LA) and Luminal B (LB) with Dunn–Šidák correction or Tukey’s HSD correction. Metabolites sorted by p-value.

No.	Metabolite	*P* value	Central tendency				Multiple comparison tests					
			HC	LA	LB	DCIS	HC vs. LA	HC vs. LB	HC vs. DCIS	LA vs. LB	DCIS vs. LA	DCIS vs. LB
1	Choline^b^	4.27E−08	0.148	0.178	0.165	0.179	+	+	+	−	−	−
2	Acetoacetate^b^	8.92E−06	0.103	0.126	0.136	0.117	+	+	+	−	−	−
3	Alanine^b^	2.28E−05	0.817	0.714	0.705	0.721	+	+	+	−	−	−
4	Citrate^b^	2.28E−05	0.066	0.072	0.086	0.078	−	+	+	−	−	−
5	Lactate^b^	9.99E−05	5.006	3.960	3.628	3.572	+	+	+	−	−	−
6	Histidine^a^	1.34E−04	0.042	0.037	0.037	0.038	+	+	−	−	−	−
7	3-Hydroxybutyrate^b^	4.03E−03	0.601	0.710	0.673	0.734	+	+	+	−	−	−
8	Tyrosine^a^	1.18E−02	0.090	0.083	0.082	0.084	+	+	−	−	−	−
9	Unk_1^b^	1.31E−02	0.117	0.107	0.104	0.106	+	+	+	−	−	−
10	Glutamate^b^	1.43E−02	0.133	0.123	0.125	0.127	+	−	−	−	−	−
11	O-Phosphocholine^a^	1.60E−02	0.398	0.426	0.408	0.420	+	−	−	−	−	−
12	Creatine^a^	3.65E−02	0152	0.158	0.151	0.173	−	−	−	−	−	+

^a^ANOVA test with Tukey’s HSD correction, ^b^Kruskal−Wallis test with Dunn–Šidák correction, Central Tendency- mean for parametric, median for non-parametric, + statistical difference between groups (< 0.05), − no statistical significance between groups (< 0.05).

### Effects of age and menopause on metabolomics and metallomics analysis

The main relative integral dataset (*N* = 318) was used to verify the most important metabolites related to menopause and age. Metabolite relative integral values were used to calculate PLS regression models in relation to subjects’ age with additional consideration of menopause status. The PLS regression models analysis was diveded into three main categories (before menopause, after menopause, combined) with three subsets (control group, breast cancer, and combined). Analysis was carried out without splitting for training and prediction to increase the sample size for particular models. Additionally, detailed univariate correlation analysis between age and menopause with metabolites’ relative integrals is presented in Supplementary Fig. S7.

**Table 7:** The PLSR models parameters and validation results for age and menopause impact.

ID	PLSR model type	LV	*N*=	*R*^2^X(cum)	*R*^2^Y(cum)	Q^2^(cum)	RMSECV	CV-ANOVA	Age range
w/o menopausal distinction									
A	HC	2	159	0.349	0.396	0.259	7.31	2.64E−09	34–82
B	BC	2	159	0.298	0.286	0.0377	9.57	3.57E−01	37–77
C	Combined	3	318	0.412	0.35	0.192	9.02	9.25E−12	34–82
Pre-menopausal									
D	HC	1	71	0.292	0.148	0.00472	4.34	8.51E−01	37–54
E	BC	1	29	0.254	0.397	0.206	5.28	5.02E−02	34–57
F	Combined	1	100	0.261	0.16	0.0678	4.70	3.32E−02	34–57
Post-menopausal									
G	HC	1	77	0.0804	0.342	− 0.1	6.52	1.00E + 00	37–77
H	BC	1	122	0.047	0.229	− 0.1	6.68	1.00E + 00	49–82
J	Combined	1	199	0.0995	0.181	0.0566	6.47	3.30E−03	37–82

The PLSR models show significant correlation (Q^2^ (cum) > 0.15, CV-ANOVA p-value < 0.05) with patient age in HC observations data (Table 7A) and in the combined dataset of HC + BC without distinction of menopausal status (Table 7C). The pre- and post-menopausal PLSR models did not exhibit significant age correlations.

**Fig. 7:**
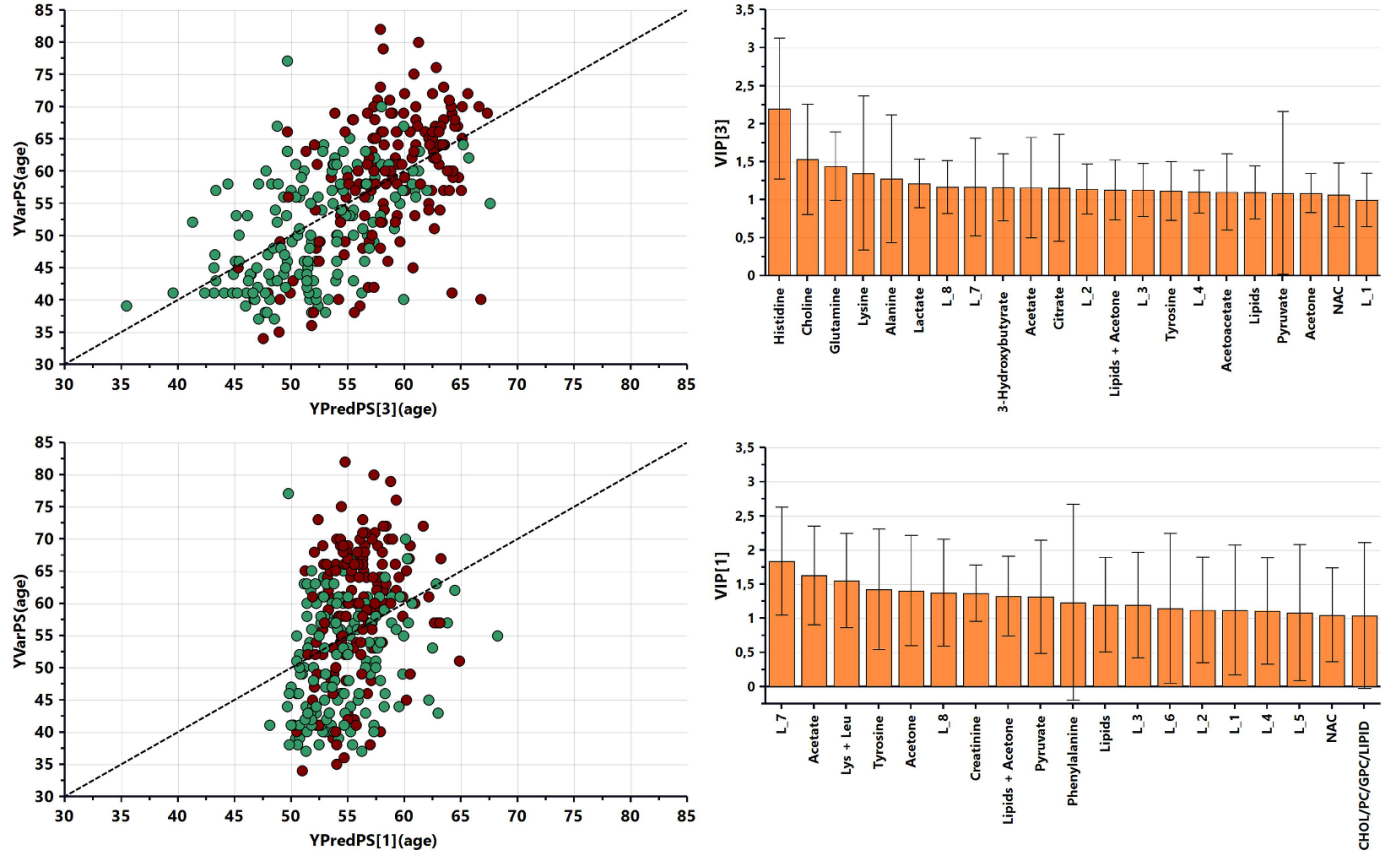
The PLS regression model for age prediction together with VIP bar plot for metabolites with VIP score > 1.00. (top) First PLSR, (bottom) Sixth iteration PLSR. Green—control group; red—breast cancer.

To extract age related relative integrals, an iterative approach was implemented for PLSR models calculation on combined data without menopausal distinction. In each iteration, the highest VIP score relative integral from the previous model was excluded until a significant decrease in R^2^ × (cum) and Q^2^(cum) was observed. The first calculated PLSR model parameters were LVs = 3, R^2^ = 0.412, Q^2^(cum) = 0.192, RMSECV = 9.02 (Table 7C) and for the sixth iteration with an excluded set of six age-related relative integrals, LVs = 1, R^2^ = 0.268, Q^2^(cum) = 0.0685, RMSECV = 9.63 (Fig. 7). The selected set of relative integrals were removed (in model iterations order) were histidine, choline, lactate, citrate, lysine, and glutamine to decrease age prediction quality in the PLSR model.

The selected set of PLSR age-related metabolites then was removed from the main dataset, and the training procedure from the methodology section was repeated. The model’s performance with removed age-related relative integrals was assessed by ROC AUC and accuracy (ACC) and presented in Table 8. Moreover, Supplementary Tables S9 and S10 represent the impact of excluding age-related relative integrals in pre and post-menopausal status observations on the discrimination analysis results between HC and BC.

**Table 8:** Average model’s performance on datasets without age related relative integrals classification threshold < 0.5.

Model type	OPLS-DA		ANN		XGB	
Dataset (training set, *N* = 238/ test set, *N* = 80)	*R*.I.	*R*.I. w/o Unknowns	*R*.I.	*R*.I. w/o Unknowns	*R*.I.	*R*.I. w/o Unknowns
Mean AUC train set (%)	0.884	0.883	0.937	0.922	0.990	0.990
Mean AUC test set (%)	0.959	0.951	0.924	0.909	0.945	0.936
Mean accuracy train set (%)	0.786	0.786	0.884	0.868	0.962	0.960
Mean accuracy test set (%)	0.875	0.863	0.839	0.822	0.863	0.857

*R.I.* Relative integrals, *AUC* Area under curve.

In retrained models, a decrease in performancewas observed. Nonetheless, models maintained an AUC above 0.9. The highest decrease was presented by ANN in both types of datasets, with (AUC_test_ = 0.924) and without unknowns (AUC_test_ = 0.909). In the case of XGB and OPLS-DA, performance for test data remained high, for XGB with unknowns AUC_test_ = 0.945 and without AUC_test_ = 0.936; and for OPLS-DA respectively AUC_test_ = 0.959 and AUC_test_ = 0.951, respectively.

**Fig. 8:**
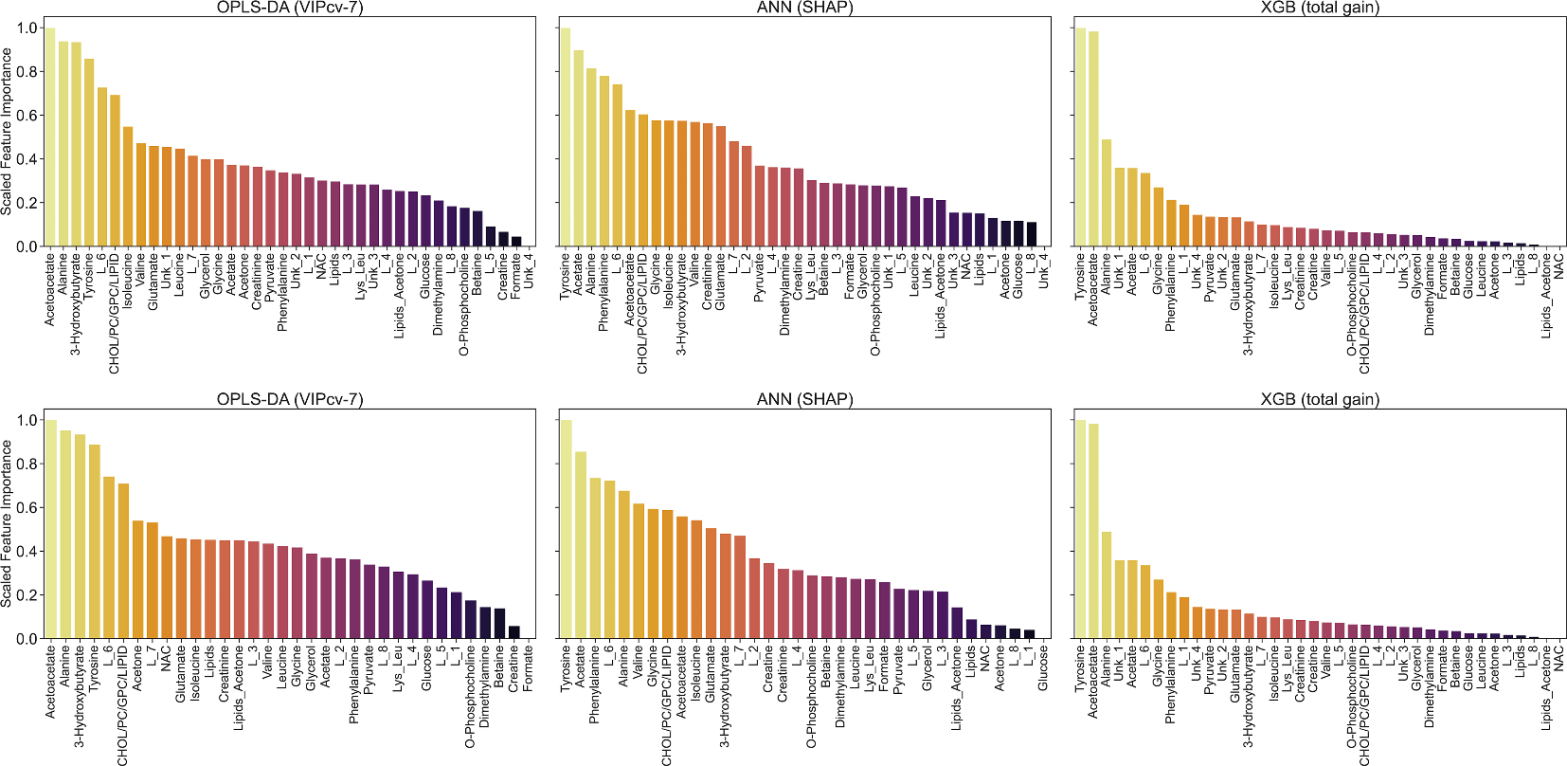
Scaled feature importance scores in dependence of model and data type trained on datasets without age-related metabolites. Top row dataset with unknown resonce singals (R.I.), bottom row, dataset without unknown resonance singals (R.I. w/o Unknowns).

In the feature importance analysis for discrimination between HC and BC on datasets without PLSR age related metabolites, the five most prominent relative integrals were selected from each model type (Fig. 8). Combined models’ feature importance analysis highlighted the following metabolites (in order of presence in the model and their rank): tyrosine, alanine, acetoacetate, acetate, L_6, 3-hydroxybutyrate, phenylalanine, L_6, and Unk_1 as the most influential across all model types (Fig. 8). The mean importance values of relative integrals for each model type are presented in Supplementary Table S10.

**Fig. 9:**
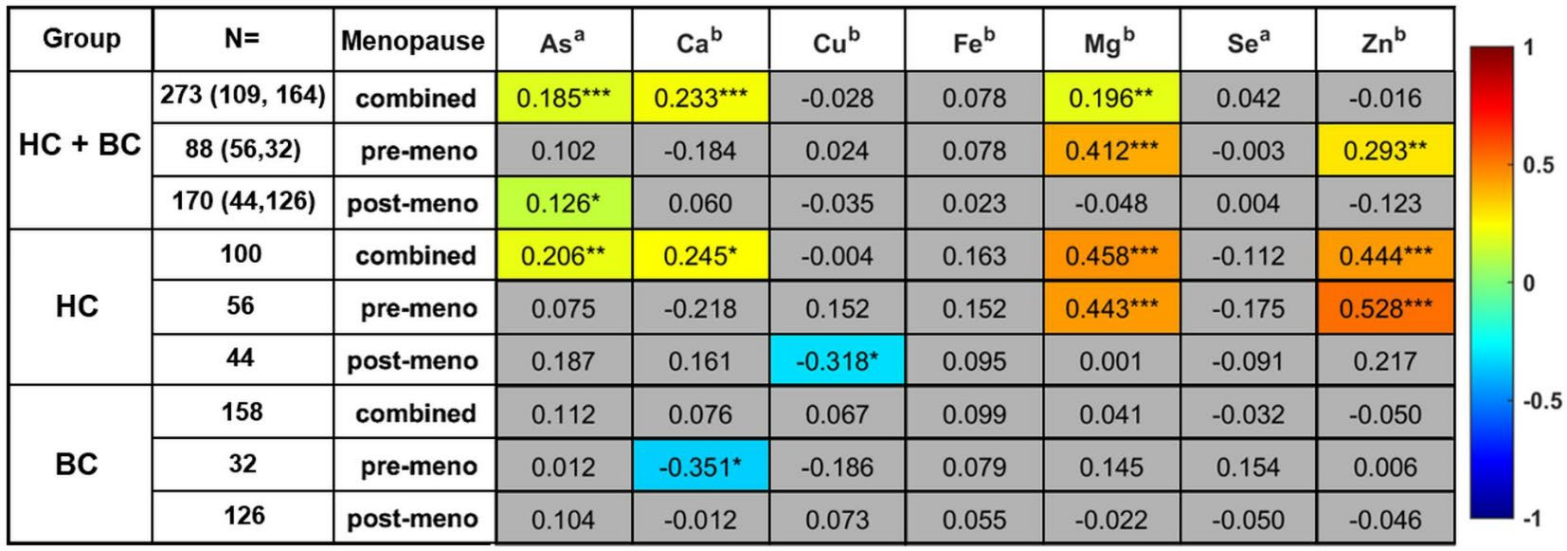
The correlation coefficients (^a^Kendall tau-b, ^b^Spearman rho) for age and metal ions concentration for specific groups depending of health and menopausal status. Grey—not statistically significant, color—statistically significant p value *< 0.05, **<0.01, ***<0.001. Color gradient corresponds to correlation coefficient.

The quantified metal ions were investigated to exclude age and menopause impacts. For this purpose, metal ion concentrations were correlated sequentially with patient age and menopausal status data. Seven statistically significant correlations between age and metal ion concentrations were observed (Fig. 9).

In combined data (HC + BC) without distinction of menopausal or health status, three statistically significant correlations were observed. Positive correlations with age were found for As, Ca, and Mg with correlation coefficients 0.185, 0.233 and 0.196, respectively (Fig. 9). Moreover, within combined data without distinction of health status, for premenopausal patients, positive correlations with age were observed for Mg (0.412) and Zn (0.293) metal ions (Fig. 9) and for postmenopausal patients, a positive correlation with age was observed for As (0.126). In the analysis of the control group data without distinction of menopausal status, four positive correlations between age and metal ions concentration were detected, namely for As, Ca, Mg, and Zn, with their corresponding correlation coefficients being 0.206, 0.245, 0.458, and 0.444, respectively. Moreover, with differentiation between pre- and post-menopausal status in the control group, significant correlations with age were observed for Mg and Zn concentrations in the premenopausal (0.443, 0.528), while in the postmenopausal group, a significant negative correlation between age and Cu was observed (− 0.318) (Fig. 9). In breast cancer samples, a single negative age correlation was observed in premenopausal patients for Ca (− 0.351).

Furthermore, Supplementary Table S13 displays a statistical analysis comparing observations of premenopausal versus postmenopausal subjects, as well as the outcomes of the control versus breast cancer comparison, with a specific focus on menopausal status. Following the FDR procedure for the comparison between pre- vs. post-menopausal statuses, statistical significance was obtained for As, Ca and Mg metal ion concentrations. Postmenopausal observations showed higher concentrations of As, Ca, and Mg compared to premenopausal observations (Supplementary Table S13).

In the analysis dependent on menopausal status, the comparison between control and breast cancer in premenopausal patients did not exhibit statistically significant differences. Conversely, the postmenopausal comparison between HC and BC revealed statistically significant differences in Se and Zn concentrations, with Se being lower and Zn higher in the control group than in the breast cancer.

## Discussion

Metabolomics analysis of breast cancer in the literature includes many quality articles combined into meaningful reviews^12,14^. None to our knowledge focuses strictly for breast cancer detection at the earliest stages through metabolomics and metallomics serum analysis. Our study primarily aimed to investigate the potential implementation of metabolomics analysis for breast cancer screening via serum samples in a reproducible manner among the studied cohort. Observations were collected to reference the naturally occurring ratio between invasive (IDC) and non-invasive breast cancer (DCIS)^1,3,45^.

The trained discrimination models for screening purposes showed, in both data types (relative integrals with and without unknown resonance signals), statistically significant discrimination with high success metrics for differentiating between control and breast cancer groups. The OPLS-DA model trained on relative integrals with unknown resonance signals achieved the highest performance across the tested dataset and model types, reaching AUC_test_ = 0.985 (Table 2). Nonetheless, a set of important features across all trained models was identified with the top five performing features (SHAP, Total gain, or VIP) being choline, tyrosine, histidine, acetoacetate, citrate, alanine, acetate, lactate and lysine (Fig. 2). Although literature reports age-related metabolites, that partially match this set of small-molecular compounds in the main screening models, they were not excluded due to treating metabolites related to patient age as a significant factor for breast cancer occurrence probability^46^. Similarly, the impact of pre- and post-menopausal status on breast cancer patients’ metabolism in response to ongoing pathological processes was considered. Nonetheless, the main trained models demonstrated stability in differentiating control from early breast cancer cohorts without removing variance from menopause/age factors. A detailed impact of age and menopause is analyzed in further section.

The univariate analysis of metabolites’ relative integrals for screening purposes (BC vs. HC) provided a set of 20 metabolites and resonance signal bins that allowedfor statistically significant discrimination (Table 3). The breast cancer group had elevated relative integrals for choline, acetoacetate, 3hydroxybutyrate, citrate, L_7, Unk_2, acetone, and glycerol (Table 3; Fig. 2) and reduced levels for histidine, tyrosine, alanine, lactate, L_6, acetate, glutamate, glycine, creatinine, CHOL/PC/GPC/LIPID, phenylalanine, valine, lysine + leucine, Unk_4, Unk_3, and pyruvate (Table 3; Fig. 3). The three most impactful and statistically important pathways based on relative integrals, linked occurring changes to phenylalanine, tyrosine and tryptophan biosynthesis, synthesis and degradation of ketone bodies and D-glutamine and D-glutamate metabolism Supplementary Table S12.

Separately, the metabolites reported in this study from univariate analysis partially overlap with small molecular compounds currently reported in the literature. However, it is important to note that only partially do their relations of increase or decrease in comparisons with the control group match published literature.

Taking into consideration two main comparisons: between control groups and the breast cancer group, and between EBC and MBC, most metabolites found in our study were identified in literature-reported studies. The statistically significant metabolites in line with the results of our studies display a visible increasing trend of 3-Hydroxybutyrate^19,35^, acetoacetate^19,35^, choline^19^, and grycerol^30^ and decreasing levels of histidine^19,30^, alanine^35^, glycine^18^, valine^18^, pyruvate^21^.

However, in other studies, metabolite levels such as acetone^24,35^, tyrosine^16,18,19^, alanine^19,21,23^, lactate^18,19,21,23,35^ acetate^19^, glutamate^18,19,21,35^, glycine^19^, creatinine^19,24^ phenylalanine^19,31,35^, valine^18,21^, and pyruvate^20^ were reported as showing an opposite trend compared to our study.

This variability across results reported in the literature could indicate high variability in study design, along with the impact of confounding factors that are not related to the organism’s response to breast cancer occurrence. Factors such as age or menopausal status, which are not isolated from analysis, may contribute to these discrepancies.

Furthermore, metal ion statistical analysis for the main BC vs. HC comparison highlighted four important ion concentrations: As, Ca, Se, and Zn. Significant differences were observed in the breast cancer group, with increased concentrations of As, Ca andSe, and decreased Zn, compared to the control cohort. Elevated arsenic concentration in serum of Polish female patients was reported in literature and connected to increased risk of breast cancer^47^. The obtained data for arsenic ICP-OES measurements in our analysis are highly left-censored, with 60% for the control group and 50% for the breast cancer group (Supplementary Table S6). The higher percentage of censored data in thr control group, together with the higher estimated median arsenic concentration for breast cancer, which was observed to be statistically significant, suggests. Suggesting an a agreement with literature reported results, connecting higher serum arsenic levels with increased breast cancer risk^47^. Increased calcium concentration in breast cancer patients was previously reported as significant for discrimination between control and breast cancer cohorts^48^ or associated with breast cancer risk in premenopausal women^49^. This association in Ca trend changes aligns with the results obtained in this study. Moreover, increased serum calcium has been reported as possibly connected to tumor-protective and promoting effects^49^. Selenium serum levels play a significant role in cellular and molecular mechanisms and are treated as an interesting prevention agent with potential treatment applications^50^. Its concentration has been positively correlated with breast cancer patient survival in multiple studies^51–53^. The selenium serum levels obtained in this study show a statistically significant concentration increase in breast cancer compared to the control cohort. However, the uncontrolled dietary intake and supplementation in our study may results in misleading conclusions, necessitating further research in a regulated environment to validate the findings. Additionally, literature reports data that contradict the observations in this study^54^. Statistically significant lower zinc levels in the breast cancer align with results from two meta-analyses^55,56^. However, it is important to recognize that current knowledge is inconsistent regarding serum zinc levels, with contradictory results reported. Some metaanalysis or recent studies highlight no significant differences in zinc serum concentration^57,58^. Nonetheless, zinc plays an important role in the cell cycle, and current trends in drug research focus on zinc transporters as possible molecular targets^59,60^.Correlation analysis results of metal ion concentrations and metabolites’ relative integrals, showed the highest number of correlations in the combined dataset without menopause distinction. Zinc concentration correlated with a subset of the most important features from MVA analysis [tyrosine (0.157), histidine (0.265), acetoacetate (0.202), alanine (0.252)], and stage progression metabolites (3HB (− 0.252)), indicating a possible important relationship between Zn and metabolism disturbances.

Discrimination between breast cancer groups in relation to the control cohort is present in the literature^12–14^. Available research studies also focuse on discrimination regardless breast cancer progression^12,13,61,62^, considering only advanced stages, combining individual stages into broader subgroups (I + II and III + IV)^18^ or focusing on amino acids composition^63,64^. The analysis provided in this study highlights dominant metabolite disturbances associated with progression in the first and second stages of breast cancer. Statistical analysis identified four low-mass compounds as key metabolites for determining progression: 3-hydroxybutyrate, acetoacetate, lysine and glycerol. However, these metabolites were not statistically significant after the FDR procedure. Reported small molecular compounds can be linked to the development of tumor volume^18,65^. Literature analysis has highlighted differences between early-stage disease (EBC, I + II) and late-stage disease (LBC, III + IV), suggesting alink between the concentration of glycerol and lysine, which may be important for monitoring progression^18^. Moreover, statistical analysis results between the stages of studied cancer integrals of low-molecular-weight compounds advocate for a connection to tumor progression, particularly metastasis processes^35^. Available literature indicates possible differences in metabolomics profiles of localized tumors and those with metastasis^35^.

### Cancer subgroups analysis

The biological material used in the studies originates from two main types of pathological changes: invasive and non-invasive. Although non-invasive lesions constituted only 21 out of 161 total observations which accounts for 13%, population-based studies report a correlation between the incidence of invasive cancers and the detection of DCIS and involved treatment before IDC diagnosis. Moreover, literature shows the possibility of a potential negative impact of over-detection of DCIS as benign lesions, which may lead to unnecessary stress for patients and, in the worst scenario, overtreatment^7,66,67^. Importantly, DCIS can be considered as a true (non-obligatory) cancer precursor, and reports reveal less common invasive lesion diagnoses when the identification of a DCIS lesion was undertaken earlier^5,68^. Identifying possible differences in the observable metabolome for HC, IDC, and DCIS could provide valuable information in the screening process. The conducted univariate analysis highlighted the partially similar impact on metabolites’ relative integrals for DCIS and IDC when compared to HC. However, in the IDC group a wider range of significant relative integrals was identified than in DCIS (Table 5). Overall, nine statistically significant compounds’ relative integrals were observed for HC vs. DCIS in multigroup comparisons, namely (in ascending p value order): histidine, tyrosine, choline, acetoacetate, alanine, 3hydroxybutyrate, citrate, lactate, and phenylalanine. All impactful relative integrals from HC vs. DCIS were also identified in HC vs. IDC, along with additional thirteen ones. Importantly, only a single metabolite’s relative integral – creatine was statistically significant for IDC vs. DCIS comparison, with a higher relative integral in IDC. Moreover, there was no statistical difference between HC and DCIS for creatine’s relative integral. In the literature creatine has been observed as a differentiation factor between benign and invasive lesions and is linked to creatine kinase functions in meeting the energetic requirements of tumor cells^69,70^.

Further subgroup analysis of invasive ductal carcinoma was conducted in our study. The IDC group had two major subgroups luminal A (LA) and luminal B (LB), which were investigated to find more precise biochemical information. The univariate analysis for comparisons between HC vs. luminal A vs. luminal B vs. DCIS identified twelve statistically significant relative integrals in multigroup comparison, leading to the selection of three non-overlapping metabolites within all investigated groups: tyrosine for HC vs. LA and HC vs. LB, citrate for HC vs. LB and HC vs. DCIS groups, and glutamate and Ophosphocholine for HC vs. LA. Finally, one unique relative integral (creatine) was statistically significant between DCIS and LB. Interestingly, detailed subgroup analysis showed that the luminal B subgroup is mainly responsible for the increased creatine relative integral in the IDC group for HC vs. IDC vs. DCIS comparison.

### Age and menopause status

Although two main sources of variability age and menopausal status were included in the study groups (HC vs. BC) for screening purposes, both factors were investigated to isolate biological variability originating from these sources. The PLSR models extracted a set of six relative integrals, which were most impactful in age prediction, namely (in order of iterative removal): histidine, choline, lactate, citrate, lysine, and glutamine. In a further step, the exclusion of these relative integrals decreased the impact of patients’ age on HC vs. BC discrimination models. Furthermore, models trained on an age-unbounded dataset enabled the identification of significant biochemical differences between control and breast cancer groups through feature importance analysis. These findings could serve as a starting point for further in-depth analysis. The extracted relative integrals across different model types were (in order of occurrence and rank): tyrosine, alanine, acetoacetate, acetate, L_6 (3.22 ppm), 3hydroxybutyrate, phenylalanine, and Unk_1 (Doublet, 1.15 ppm) (Table 3 and Supplementary S10). Additionally, without age-related relative integrals, the predictive performance of the trained models for breast cancer screening remained high (Table 8), where for the best performing model type—OPLSDA AUC_test_ was equal to 0.959. Collectively, the relative integrals of tyrosine, alanine, L_6, phenylalanine, acetate, and Unk_1 decreased, while acetoacetate, and 3-hydroxybutyrate increased in the studied breast cancer samples. In analyzing the impact of menopause on the best performing in screening model type (OPLS-DA), the dataset was split into pre- and post-menopausal observations, and then HC vs. BC discrimination was conducted, resulting in an AUC_test_ above 0.900 (Supplementary Table S9). However, in the case of premenopausal models, Q^2^(cum) were below 0.2. Additionally, in datasets without unknown resonance signals, the premenopausal model was not statistically significant in CV-ANOVA (*p* < 0.05) (Supplementary Table S9). Importantly, although the trained models obtained high AUC_test_ values, these analyses were carried out on unbalanced data with lower sample sizes than the main screening model or age-unbounded HC vs. BC models. Finally, OPLS discriminant analysis between pre- vs. post-menopausal patients, without distinction of disease status based on a dataset without age-related relative integrals, showed a major increase in VIP values of quantified lipids fragments. However, these findings need to be taken with caution due to unsatisfactory Q^2^(cum) values, and lower AUC_test_ (R.I. = 0.718, R.I. w/o unknowns = 0.687), decreasing the possibility of drawing statistically meaningful information (Supplementary Table S8).

Concerning the analysis of age and menopausal status with metal ion concentration, the obtained results indicated that important differences in the studied cohorts could be only partially be associated with breast cancer. Among important metal ions, selenium concentrations did not show any significant correlation with age or important relative integrals selected in MVA. Nonetheless, selenium concentration was important in discriminating control and breast cancer patients for combined (pre- and post-menopausal) and post-menopausal data (Fig. 9, Supplementary Table S13). Other important metal ions in control and breast cancer discrimination—As, Ca and Zn—exhibited correlations with age and/or menopausal status due to overlapping importance in-between comparisons (Fig. 9, Supplementary Table S13). The arsenic correlation with age could align with possible organism accumulation and an increased risk of breast cancer, which could explain higher levels of arsenic in the breast cancer group due to the age factor^47^. The Ca concentration was observed to correlate with age and revealed no statistical significance in comparisons for pre- or post-menopausal observations for discrimination between control and breast cancer. Moreover, correlations between Ca concentration and a subset of age-related metabolites were observed, particularly, in datasets without menopause distinction, where in combined (HC + BC) data, Ca correlated with citrate relative integral (0.173), and in control observations, Ca correlated with citrate (0.285) and choline (0.231). In the case of Zn concentration, a statistically significant correlation with age was observed in pre-menopausal combined (HC + BC) data (0.293) and samples originating from the control group without distinction of menopausal status (0.444) and a subgroup of pre-menopausal (0.528). No significant correlations for Zn were observed in overall breast cancer observations. Moreover, zinc concentration correlated with a subset of the most important metabolites in age- unbounded feature importance analysis (tyrosine, alanine, acetoacetate, 3HB). Finally, the analysis results indicated the importance of Se and Zn in the discrimination of control and breast cancer groups, however, these findings need further investigation due to the possible significant influence of menopausal status and supplementation. In the case of Mg, significant age correlations were observed in pre-menopausal and without menopausal distinction HC + BC data. Analogous correlations were found for subgroups in control samples. Moreover, comparable to other metal ions, no significant correlations in breast cancer samples were observed. Furthermore, Mg-age correlations and differences between pre- and post-menopausal groups were confirmed in univariate statistics (Supplementary Table S13).

Even though copper concentration was not important for breast cancer discrimination, the obtained results showed significant correlation between a vast number of quantified resonance signals. The correlation analysis for Cu concentration showed significant and strong correlations among most of the quantified resonance signals in the premenopausal control group. The concentration of Cu metal ions was observed to have a statistically significant positive correlation with 18 relative integrals and 11 negative correlations among quantified resonance signals (Supplementary Fig. S6). This showed differences in metabolism between pre- and post-menopausal patients; moreover, this pattern of correlation was not observed in breast cancer samples (Supplementary Fig. S6).

## Conclusion

The obtained results of the study highlight the discriminative ability of metabolomics and metallomics tools in breast cancer screening and show their potential application. Additionally, the analysis of the impact of age and menopause factors demonstrated that the metabolomics approach can be applied to screening despite co-occurrence other important factors. Data collected from NMR and ICP-EOS enabled univariate analysis and feature importance analysis across different data subsets and MVA models, providing sets of relative integrals and metal ion concentrations related to breast cancer response and their subtypes, markers of progression, age and menopause. Furthermore, investigating the impact of age and menopause clarified important features unrelated to the breast cancer biochemical response while highlighting variables related to these factors.

## Electronic supplementary material

Below is the link to the electronic supplementary material.

Supplementary Material 1

## Data Availability

The datasets generated during and/or analyzed during the current study are available from the corresponding author upon request.
