# Supplementary material for: Serum metabolite and metal ions profiles for breast cancer screening
Source: Sci Rep. 2024 Oct 19;14:24559. doi: 10.1038/s41598-024-73097-1 (PMC11490637; doi:10.1038/s41598-024-73097-1)
Supplement: Supplementary file 1 — Supplementary Material 1 [file 41598_2024_73097_MOESM1_ESM.docx]

**SUPPLEMENTARY DATA**

Table S 1. Medical information of control group enrolled in metabolomics analysis.

| **Control group (HC)** | **Average age** | **Range** | **34-43** | **44-53** | **54-63** | **64-73** | **74-83** |
| --- | --- | --- | --- | --- | --- | --- | --- |
| N= | 51.2 | 37-77 | 39 | 54 | 59 | 8 | 1 |
| **Menopausal status** | **Pre-menopausal** | **Post-menopausal** | **N.D** |  |  |  |  |
| N= | 72 | 78 | 11 |  |  |  |  |
| **Comorbidity** | **Diabetes** | **Hypertension** | **Hypothyroidism** | **Hyperthyroidism** | **N.D.** |  |  |
| N= | 8 | 40 | 21 | 0 | 3 |  |  |
| **Smoking** | **Smokers** | **Non-smokers** | **N.D.** |  |  |  |  |
| N= | 37 | 118 | 6 |  |  |  |  |

Table S 2. Medical information of breast cancer patients enrolled in metabolomics analysis.

| **Average age** | **Range** | **34-43** | **44-53** | **54-63** | **64-73** | **74-83** |
| --- | --- | --- | --- | --- | --- | --- |
| 59.4 | 34-82 | 16 | 23 | 58 | 59 | 5 |
| **IDC** | **DCIS** |  |  |  |  |  |
| 140 | 21 |  |  |  |  |  |
| **Luminal A** | **Luminal B** | **TNBC** | **DCIS** | **Non-luminal** | **N.D.** |  |
| 63 | 46 | 9 | 21 | 3 | 19 |  |
| **I** | **II** | **III** | **N.D.** |  |  |  |
| 70 | 59 | 4 | 28 |  |  |  |
| **Her2+** | **Borderline** | **Her2-** | **N.D.** |  |  |  |
| 18 | 27 | 79 | 37 |  |  |  |
| **ER+** | **ER-** | **N.D.** |  |  |  |  |
| 124 | 19 | 18 |  |  |  |  |
| **PgR+** | **PgR-** | **N.D.** |  |  |  |  |
| 113 | 28 | 20 |  |  |  |  |
| **Low** | **Intermediate** | **High** | **N.D.** |  |  |  |
| 70 | 11 | 38 | 42 |  |  |  |
| **Pre-menopausal** | **Post-menopausal** | **N.D** |  |  |  |  |
| 29 | 124 | 8 |  |  |  |  |
| **Diabetes** | **Hypertension** | **Hypothyroidism** | **Hyperthyroidism** | **N.D.** |  |  |
| 11 | 56 | 12 | 13 | 5 |  |  |

*The high Ki-67 expression were classified as ≥ 20%, intermediate 19% - 14% and those with < 14% as low Ki‑67 expression.

Table S 3. Datasets split based on Kennard-Stone algorithm and theirs basic medical and demographical information.

| **Dataset** | **Training** | | **Test** | |
| --- | --- | --- | --- | --- |
| **Group** | **HC** | **BC** | **HC** | **BC** |
| **Average** | 51.21 | 58.96 | 51.05 | 60.85 |
| **SD** | 8.47 | 10.07 | 8.57 | 8.38 |
| **IDC** | - | 103 | - | 37 |
| **DCIS** | - | 18 | - | 3 |
| **Luminal A** | - | 43 | - | 20 |
| **Luminal B** | - | 35 | - | 11 |
| **TNBC** | - | 9 | - | 0 |
| **Non-Luminal** | - | 2 | - | 1 |
| **N.D.** | - | 14 | - | 5 |
| **Numbers per group** | 121 | 121 | 40 | 40 |

Table S 4**.** Medical information of control group enrolled in metal ions analysis.

| **Control group (HC)** | **Average age** | **Range** | **20-43** | **44-53** | **54-63** | **64-73** | **74-83** |
| --- | --- | --- | --- | --- | --- | --- | --- |
| N= 109 | 45.73 | 20-67 | 41 | 26 | 34 | 8 | 0 |
| **Menopausal status** | **Pre-menopausal** | **Post-menopausal** | **N.D** |  |  |  |  |
| N= | 56 | 44 | 9 |  |  |  |  |
| **Comorbidity** | **Diabetes** | **Hypertension** | **Hypothyroidism** | **Hyperthyroidism** | **N.D.** |  |  |
| N= | 8 | 18 | 13 | 0 | 56 |  |  |
| **Smoking** | **Smokers** | **Non-smokers** | **N.D.** |  |  |  |  |
| N= | 21 | 85 | 3 |  |  |  |  |

Table S 5. Medical information of breast cancer patients enrolled in metal ions analysis

| **Breast cancer (BC)** | **Average age** | **Range** | **34-43** | **44-53** | **54-63** | **64-73** | **74-83** |
| --- | --- | --- | --- | --- | --- | --- | --- |
| N= | 59.18 | 32-82 | 16 | 22 | 56 | 63 | 7 |
| **Type** | **IDC** | **DCIS** |  |  |  |  |  |
| N= | 143 | 21 |  |  |  |  |  |
| **Subtype** | **Luminal A** | **Luminal B** | **TNBC** | **DCIS** | **Non-luminal** | **N.D.** |  |
| N= | 61 | 48 | 11 | 21 | 3 | 20 |  |
| **Stage** | **I** | **II** | **III** | **N.D.** |  |  |  |
| N= | 71 | 57 | 6 | 30 |  |  |  |
| **HER2 status** | **Her+** | **Borderline** | **Her-** | **N.D.** |  |  |  |
| N= | 18 | 28 | 80 | 38 |  |  |  |
| **ER status** | **ER+** | **ER-** | **N.D.** |  |  |  |  |
| N= | 125 | 20 | 19 |  |  |  |  |
| **PgR status** | **PgR+** | **PgR-** | **N.D.** |  |  |  |  |
| N= | 112 | 31 | 21 |  |  |  |  |
| **Ki-67 level*** | **Low** | **Intermediate** | **High** | **N.D.** |  |  |  |
| N= | 69 | 12 | 39 | 44 |  |  |  |
| **Menopausal status** | **Pre-menopausal** | **Post-menopausal** | **N.D** |  |  |  |  |
| N= | 32 | 126 | 6 |  |  |  |  |

*The high Ki-67 expression were classified as ≥ 20%, intermediate 19% - 14% and those with < 14% as low Ki‑67 expression.

Table S 6. The percentage values below limit of detection (LOD) for metal ions concentration.

| **Metal ions** | **Values <LOD for HC [%]** | **Values <LOD for BC [%]** |
| --- | --- | --- |
| **As** | 60.55 | 50.00 |
| **Ca** | 0.00 | 0.00 |
| **Cd** | 87.16 | 86.59 |
| **Co** | 73.39 | 87.20 |
| **Cr** | 65.14 | 70.12 |
| **Cu** | 0.00 | 0.00 |
| **Fe** | 0.00 | 0.00 |
| **Mg** | 0.00 | 0.00 |
| **Ni** | 76.15 | 77.44 |
| **Pb** | 88.07 | 81.10 |
| **Se** | 29.36 | 18.90 |
| **Zn** | 0.00 | 0.00 |

Table S 7**.** Metabolites assignments with observed chemical shift of signals used for calculations of relative integral along with HMDB ID.

| **No.** | **Signal assignment** | **Chemical shift** | **Identification level** | **HMDB ID** |
| --- | --- | --- | --- | --- |
| 1 | L_1 (LDL/VLDL)* | 0.85 ppm | 3 | - |
| 2 | Leucine | 0.96 ppm |  | HMDB0000687 |
| 3 | Valine | 0.99 ppm | 2 | HMDB0000883 |
| 4 | Isoleucine | 1.02 ppm | 2 | HMDB0000172 |
| 5 | Unk_1 (Doublet) | 1.15 ppm | 4 | - |
| 6 | Unk_2 (Singlet) | 1.18 ppm | 4 | - |
| 7 | 3-Hydroxybutyrate | 1.20 ppm | 2 | HMDB0000357 |
| 8 | L_2 (LDL/VLDL)* | 1.28 ppm | 3 | - |
| 9 | Lactate | 1.33 ppm | 2 | HMDB0000190 |
| 10 | Alanine | 1.48 ppm | 2 | HMDB0000161 |
| 11 | Lys + L_3 (Adipic acid) | 1.58 ppm | 3 | - |
| 12 | Lys + Leu | 1.71 ppm | 3 | - |
| 13 | Lysine | 1.89 ppm | 2 | HMDB0000182 |
| 14 | Acetate | 1.92 ppm | 2 | HMDB0000042 |
| 15 | L_4 (CH2C=C) | 2.01 ppm | 3 | - |
| 16 | NAC (N-acetylated compounds) | 2.04 ppm | 3 | - |
| 17 | Glutamine | 2.12 ppm | 2 | HMDB0000641 |
| 18 | Lipids + Acetone | 2.21 ppm | 3 | - |
| 19 | Acetone | 2.23 ppm | 2 | HMDB0001659 |
| 20 | Lipids | 2.25 ppm | 3 | - |
| 21 | Acetoacetate | 2.28 ppm | 2 | HMDB0000060 |
| 22 | Glutamate | 2.34 ppm | 2 | HMDB0003339 |
| 23 | Pyruvate | 2.37 ppm | 2 | HMDB0000243 |
| 24 | Citrate | 2.53 ppm | 2 | HMDB0000094 |
| 25 | Dimethylamine | 2.72 ppm | 2 | HMDB0000087 |
| 26 | L_5 (C=CCH2C=C) | 2.75 ppm | 3 | - |
| 27 | Creatine | 3.04 ppm | 2 | HMDB0000064 |
| 28 | Creatinine | 3.05 ppm | 2 | HMDB0000562 |
| 29 | Unk_3 (Singlet) | 3.14 ppm | 4 | - |
| 30 | Unk_4 (Singlet) | 3.15 ppm | 4 | - |
| 31 | Choline | 3.2 ppm | 2 | HMDB0000097 |
| 32 | O-Phosphocholine | 3.21 ppm | 2 | HMDB0001565 |
| 33 | L_6 | 3.22 ppm | 3 | - |
| 34 | Chol/PC/GPC/LIPID | 3.17 ppm | 3 | - |
| 35 | Glucose | 3.25 ppm | 2 | HMDB0000122 |
| 36 | Betaine | 3.27 ppm | 2 | HMDB0000043 |
| 37 | Glycine | 3.56 ppm | 2 | HMDB0000123 |
| 38 | Glycerol | 3.58 ppm | 2 | HMDB0000131 |
| 39 | L_7 (glycerol of lipids) | 5.19 ppm | 3 | - |
| 40 | L_8 (poly-UFA/fatty acyl groups) | 5.30 ppm | 3 | - |
| 41 | Tyrosine | 6.90 ppm | 2 | HMDB0000158 |
| 42 | Histidine | 7.04 ppm | 2 | HMDB0000177 |
| 43 | Phenylalanine | 7.32 ppm | 2 | HMDB0000159 |
| 44 | Formate | 8.46 ppm | 2 | HMDB0000142 |

* LDL-low density lipid; VLDL- very low density lipid

Table S 8. Variables importance mean values for each model type trained in study.

| Model Type | OPLS-DA (VIPcv-7) | | ANN (SHAP) | | XGB (total gain) | |
| --- | --- | --- | --- | --- | --- | --- |
| **Dataset** | **R.I.** | **R.I. w/o Unknowns** | **R.I.** | **R.I. w/o Unknowns** | **R.I.** | **R.I. w/o Unknowns** |
| 3-Hydroxybutyrate | 1.49918 | 1.44038 | 0.006484 | 0.008394 | 2.489129 | 2.804982 |
| Acetate | 0.821852 | 0.789767 | 0.008507 | 0.013068 | 12.04401 | 15.78228 |
| Acetoacetate | 1.56538 | 1.51656 | 0.006717 | 0.008975 | 33.42669 | 35.80589 |
| Acetone | 0.964876 | 0.932636 | 0.002141 | 0.002687 | 1.952115 | 2.209067 |
| Alanine | 1.51038 | 1.45228 | 0.008999 | 0.010493 | 24.6384 | 25.70376 |
| Betaine | 0.651862 | 0.650063 | 0.002748 | 0.003685 | 1.517245 | 1.948483 |
| CHOL/PC/GPC/LIPID | 1.04006 | 1.01746 | 0.007747 | 0.01042 | 8.749737 | 11.00584 |
| Choline | 1.66864 | 1.61697 | 0.011459 | 0.014805 | 88.06887 | 84.58385 |
| Citrate | 1.45651 | 1.41277 | 0.01117 | 0.015352 | 30.9032 | 32.93103 |
| Creatine | 0.29891 | 0.307837 | 0.005712 | 0.008039 | 5.670539 | 5.174641 |
| Creatinine | 0.845309 | 0.824596 | 0.00651 | 0.006708 | 4.42121 | 4.392074 |
| Dimethylamine | 0.290364 | 0.289852 | 0.003288 | 0.004626 | 2.496969 | 2.985311 |
| Formate | 0.141765 | 0.1338 | 0.003563 | 0.005196 | 1.251183 | 2.380609 |
| Glucose | 0.48794 | 0.471441 | 0.002018 | 0.002522 | 5.145109 | 5.053921 |
| Glutamate | 0.975472 | 0.937962 | 0.004979 | 0.006381 | 4.284828 | 4.950923 |
| Glutamine | 0.747337 | 0.721396 | 0.003535 | 0.004124 | 8.558387 | 8.849738 |
| Glycerol | 0.642808 | 0.619184 | 0.004425 | 0.005945 | 6.028152 | 5.682896 |
| Glycine | 0.659886 | 0.649315 | 0.007696 | 0.010789 | 12.7063 | 15.82017 |
| Histidine | 1.65083 | 1.58852 | 0.011284 | 0.013476 | 58.04887 | 55.09215 |
| Isoleucine | 0.752026 | 0.737503 | 0.006379 | 0.008202 | 3.445389 | 3.304703 |
| L_1 | 1.02238 | 0.992164 | 0.001803 | 0.002353 | 5.724558 | 5.779794 |
| L_2 | 1.13691 | 1.09826 | 0.004158 | 0.005851 | 1.622841 | 1.384506 |
| L_3 | 1.0789 | 1.03906 | 0.002294 | 0.003051 | 1.189008 | 1.374201 |
| L_4 | 1.12023 | 1.08379 | 0.00407 | 0.005455 | 1.105752 | 1.216688 |
| L_5 | 0.869857 | 0.847434 | 0.002706 | 0.00301 | 1.094824 | 1.289369 |
| L_6 | 1.10308 | 1.08377 | 0.008579 | 0.011247 | 22.8659 | 22.82782 |
| L_7 | 0.824904 | 0.806209 | 0.006576 | 0.007342 | 6.007336 | 6.169911 |
| L_8 | 1.04825 | 1.01329 | 0.001603 | 0.001992 | 0.925706 | 1.378876 |
| Lactate | 1.34855 | 1.30755 | 0.006772 | 0.009333 | 31.55964 | 32.55656 |
| Leucine | 0.877912 | 0.848223 | 0.002767 | 0.004159 | 2.825148 | 3.525901 |
| Lipids | 1.05206 | 1.01545 | 0.001932 | 0.002325 | 2.233939 | 2.191163 |
| Lipids + Acetone | 1.03353 | 0.998041 | 0.002048 | 0.002824 | 1.025765 | 1.925146 |
| Lys + Leu | 1.00972 | 0.983883 | 0.004626 | 0.006578 | 5.478662 | 6.043974 |
| Lysine | 0.69896 | 0.681734 | 0.006563 | 0.00935 | 12.05914 | 12.33059 |
| NAC | 0.907018 | 0.875927 | 0.002911 | 0.004089 | 1.79963 | 2.723046 |
| O-Phosphocholine | 0.704787 | 0.657317 | 0.003382 | 0.004678 | 1.317853 | 1.720755 |
| Phenylalanine | 0.948385 | 0.909992 | 0.008067 | 0.010367 | 12.76206 | 10.98778 |
| Pyruvate | 0.730354 | 0.702245 | 0.002852 | 0.003488 | 4.162998 | 5.627115 |
| Tyrosine | 1.50398 | 1.44627 | 0.011607 | 0.016756 | 53.82256 | 67.5347 |
| Unk_1 | 0.811954 | - | 0.003441 | - | 14.53986 | - |
| Unk_2 | 0.603794 | - | 0.003018 | - | 2.951028 | - |
| Unk_3 | 0.490151 | - | 0.003042 | - | 4.896815 | - |
| Unk_4 | 0.036027 | - | 0.000607 | - | 10.16167 | - |
| Valine | 1.06984 | 1.03314 | 0.006964 | 0.00928 | 3.679456 | 3.364279 |

Table S 9. Models parameters for OPLS-DA in dependence of menopausal status without variables related to age in PLSR.

| **Comparison** | **Dataset** | **Menopausal status** | **LVs** | **N** | **R2X(cum)** | **R2Y(cum)** | **Q2(cum)** | **CV-ANOVA p value** | **AUC_test_** |
| --- | --- | --- | --- | --- | --- | --- | --- | --- | --- |
| **HC vs BC** | **w/o age related, with Unknown** | **Pre-meno** | 1+1+0 | 78 | 0.377 | 0.419 | 0.125 | 4.24E-02 | 0.955 |
|  |  | **Post-meno** | 1+2+0 | 146 | 0.415 | 0.564 | 0.435 | 2.88E-15 | 0.949 |
|  | **w/o age related, w/o Unknowns** | **Pre-meno** | 1+2+0 | 78 | 0.455 | 0.515 | 0.128 | 1.25E-01 | 0.904 |
|  |  | **Post-meno** | 1+2+0 | 146 | 0.45 | 0.556 | 0.419 | 1.89E-14 | 0.946 |
| **Pre vs Post menopause** | **w/o age related, with Unknown** | **N.A.** | 1+0+0 | 224 | 0.265 | 0.105 | 0.0568 | 1.56E-03 | 0.718 |
|  | **w/o age related, w/o Unknowns** | **N.A.** | 1+1+0 | 224 | 0.404 | 0.180 | 0.0807 | 9.85E-04 | 0.687 |

Table S 10. Cross-validated (7-fold) variable importance in projection for OPLS-DA in dependence of menopausal status without variables related to age in PLSR.

| **Dataset** | **w/o age related, with Unknown** | | **w/o age related, w/o Unknowns** | |
| --- | --- | --- | --- | --- |
|  | **Pre-meno** | **Post-meno** | **Pre-meno** | **Post-meno** |
| **3-Hydroxybutyrate** | 0.612055 | 1.68076 | 0.561819 | 1.61153 |
| **Acetate** | 0.652364 | 0.804461 | 0.680186 | 0.802257 |
| **Acetoacetate** | 0.889344 | 1.80551 | 0.736141 | 1.76089 |
| **Acetone** | 1.31736 | 1.01642 | 1.19407 | 0.982345 |
| **Alanine** | 0.676515 | 1.70001 | 0.714024 | 1.65026 |
| **Betaine** | 0.554975 | 0.609873 | 0.717214 | 0.580576 |
| **CHOL/PC/GPC/LIPID** | 1.91302 | 1.03316 | 1.73144 | 0.98978 |
| **Creatine** | 0.713738 | 0.364258 | 0.569934 | 0.369554 |
| **Creatinine** | 1.02866 | 0.65238 | 1.11358 | 0.693064 |
| **Dimethylamine** | 0.74944 | 0.47953 | 0.558865 | 0.462018 |
| **Formate** | 0.273902 | 0.542538 | 0.484108 | 0.518304 |
| **Glucose** | 0.699246 | 0.571669 | 0.608175 | 0.541031 |
| **Glutamate** | 0.20383 | 1.11503 | 0.314752 | 1.1111 |
| **Glycerol** | 0.401907 | 0.979105 | 0.951546 | 0.949201 |
| **Glycine** | 0.630673 | 1.02221 | 0.679148 | 1.04464 |
| **Isoleucine** | 0.135768 | 0.570316 | 0.475137 | 0.556938 |
| **L_1** | 1.26966 | 1.28184 | 1.10086 | 1.23927 |
| **L_2** | 1.18354 | 1.29522 | 1.08726 | 1.24815 |
| **L_3** | 1.1722 | 1.17086 | 1.07259 | 1.11811 |
| **L_4** | 1.19045 | 1.41178 | 1.08469 | 1.37141 |
| **L_5** | 0.997453 | 0.875918 | 1.00459 | 0.849012 |
| **L_6** | 2.01687 | 1.10581 | 1.82703 | 1.06328 |
| **L_7** | 0.787685 | 1.08734 | 0.808082 | 1.06629 |
| **L_8** | 1.14137 | 1.23145 | 1.06797 | 1.1901 |
| **Leucine** | 0.478273 | 0.547993 | 0.401143 | 0.544641 |
| **Lipids** | 1.15336 | 1.09162 | 1.04348 | 1.04299 |
| **Lipids + Acetone** | 1.21105 | 1.08821 | 1.09263 | 1.04489 |
| **Lys + Leu** | 0.884824 | 0.564109 | 0.916923 | 0.560199 |
| **NAC** | 1.2777 | 0.946653 | 1.20536 | 0.910973 |
| **O-Phosphocholine** | 0.70744 | 0.56227 | 0.674734 | 0.571144 |
| **Phenylalanine** | 1.12939 | 0.717127 | 1.47203 | 0.729955 |
| **Pyruvate** | 0.793465 | 0.180651 | 0.799585 | 0.171412 |
| **Tyrosine** | 1.75458 | 1.53599 | 1.92088 | 1.5284 |
| **Unk_1** | 1.0117 | 1.26266 | - | - |
| **Unk_2** | 0.857851 | 0.54365 | - | - |
| **Unk_3** | 0.993466 | 0.408931 | - | - |
| **Unk_4** | 0.324328 | 0.346087 | - | - |
| **Valine** | 0.607454 | 0.510576 | 0.713203 | 0.501151 |

Table S 11. Variables importance mean values for each model type in study trained without variables related to age.

| Model Type | OPLS-DA (VIPcv-7) | | ANN (SHAP) | | XGB (total gain) | |
| --- | --- | --- | --- | --- | --- | --- |
| **Dataset** | **R.I.** | **R.I. w/o Unknowns** | **R.I.** | **R.I. w/o Unknowns** | **R.I.** | **R.I. w/o Unknowns** |
| 3-Hydroxybutyrate | 2.03722 | 1.8428 | 0.006761 | 0.007061 | 10.42808 | 10.85232 |
| Acetate | 0.833514 | 0.765615 | 0.010239 | 0.01132 | 27.22569 | 38.98232 |
| Acetoacetate | 2.17657 | 1.96783 | 0.007286 | 0.007953 | 70.30787 | 81.90569 |
| Acetone | 0.827769 | 1.08926 | 0.001839 | 0.002295 | 4.137399 | 3.375692 |
| Alanine | 2.04575 | 1.87898 | 0.009345 | 0.009286 | 36.26168 | 37.85478 |
| Betaine | 0.381212 | 0.32306 | 0.003706 | 0.004839 | 4.916659 | 7.206647 |
| CHOL/PC/GPC/LIPID | 1.51818 | 1.41375 | 0.007081 | 0.008283 | 6.940269 | 7.713426 |
| Creatine | 0.176571 | 0.16775 | 0.004419 | 0.005523 | 8.087266 | 8.257951 |
| Creatinine | 0.813988 | 0.918143 | 0.006642 | 0.00522 | 8.391586 | 11.23329 |
| Dimethylamine | 0.483383 | 0.333788 | 0.00446 | 0.004788 | 5.457583 | 7.055092 |
| Formate | 0.129239 | 0.054921 | 0.003631 | 0.004537 | 5.069954 | 4.079418 |
| Glucose | 0.535307 | 0.565538 | 0.001838 | 0.001579 | 4.230339 | 4.782033 |
| Glutamate | 1.01857 | 0.936286 | 0.006502 | 0.007335 | 11.7003 | 15.10027 |
| Glycerol | 0.889806 | 0.80242 | 0.003584 | 0.004081 | 6.130638 | 6.857526 |
| Glycine | 0.887427 | 0.85544 | 0.006784 | 0.008344 | 21.17658 | 24.8804 |
| Isoleucine | 1.20846 | 0.924067 | 0.006773 | 0.007756 | 9.226293 | 12.68162 |
| L_1 | 0.708847 | 0.464893 | 0.001978 | 0.002052 | 15.59621 | 12.88921 |
| L_2 | 0.571313 | 0.759332 | 0.005537 | 0.005773 | 6.416942 | 9.293818 |
| L_3 | 0.642267 | 0.907802 | 0.003681 | 0.004038 | 3.724544 | 3.845611 |
| L_4 | 0.589752 | 0.621459 | 0.00448 | 0.005159 | 6.688741 | 8.493689 |
| L_5 | 0.228475 | 0.505128 | 0.003477 | 0.004121 | 7.49201 | 9.550349 |
| L_6 | 1.59156 | 1.47538 | 0.008557 | 0.009824 | 25.6814 | 30.41498 |
| L_7 | 0.922435 | 1.07378 | 0.005759 | 0.006946 | 9.427193 | 12.43361 |
| L_8 | 0.427283 | 0.688014 | 0.001774 | 0.002118 | 3.140484 | 3.508412 |
| Leucine | 0.992655 | 0.868004 | 0.003046 | 0.004707 | 4.164744 | 4.379425 |
| Lipids | 0.669908 | 0.92183 | 0.0022 | 0.002596 | 3.541852 | 4.021237 |
| Lipids_Acetone | 0.576323 | 0.917549 | 0.002861 | 0.003228 | 2.468875 | 2.707066 |
| Lys_Leu | 0.641107 | 0.644094 | 0.003841 | 0.004698 | 8.633379 | 8.329324 |
| NAC | 0.679544 | 0.953806 | 0.002234 | 0.002317 | 2.430404 | 2.766835 |
| O-Phosphocholine | 0.411066 | 0.392323 | 0.003574 | 0.004884 | 7.020766 | 7.500167 |
| Phenylalanine | 0.758935 | 0.751122 | 0.008966 | 0.009951 | 17.21327 | 20.88854 |
| Pyruvate | 0.77983 | 0.705056 | 0.004555 | 0.004183 | 11.93047 | 12.00362 |
| Tyrosine | 1.87355 | 1.75421 | 0.01133 | 0.012956 | 71.43155 | 68.13047 |
| Unk_1 | 1.00982 | - | 0.003539 | - | 27.29463 | - |
| Unk_2 | 0.745578 | - | 0.002961 | - | 11.77967 | - |
| Unk_3 | 0.641043 | - | 0.002243 | - | 6.182804 | - |
| Unk_4 | 0.029883 | - | 0.000563 | - | 12.51482 | - |
| Valine | 1.04474 | 0.889047 | 0.006705 | 0.008615 | 7.654233 | 7.154906 |

Table S 12. Detailed results from the pathway analysis.

|  | Total Cmpd | Hits | Raw p | -LOG10(p) | Holm adjust | FDR | Impact |
| --- | --- | --- | --- | --- | --- | --- | --- |
| Phenylalanine, tyrosine and tryptophan biosynthesis | 4 | 2 | 4.52E-22 | 21.344 | 1.45E-20 | 3.85E-21 | 1 |
| Synthesis and degradation of ketone bodies | 5 | 2 | 3.07E-13 | 12.513 | 7.06E-12 | 8.69E-13 | 0.6 |
| D-Glutamine and D-glutamate metabolism | 6 | 2 | 7.98E-05 | 4.0978 | 0.000958 | 0.000109 | 0.5 |
| Phenylalanine metabolism | 10 | 2 | 4.52E-22 | 21.344 | 1.45E-20 | 3.85E-21 | 0.35714 |
| Alanine, aspartate and glutamate metabolism | 28 | 5 | 7.20E-21 | 20.143 | 2.09E-19 | 4.08E-20 | 0.3109 |
| Glycine, serine and threonine metabolism | 33 | 5 | 3.61E-17 | 16.442 | 9.40E-16 | 1.37E-16 | 0.29611 |
| Pyruvate metabolism | 22 | 3 | 9.98E-07 | 6.001 | 1.60E-05 | 1.79E-06 | 0.26749 |
| Glycerolipid metabolism | 16 | 1 | 0.027258 | 1.5645 | 0.16355 | 0.030893 | 0.23676 |
| Histidine metabolism | 16 | 2 | 4.59E-20 | 19.338 | 1.29E-18 | 2.23E-19 | 0.22131 |
| Tyrosine metabolism | 42 | 3 | 2.12E-25 | 24.674 | 6.99E-24 | 3.60E-24 | 0.13972 |
| Glyoxylate and dicarboxylate metabolism | 32 | 7 | 6.25E-13 | 12.204 | 1.37E-11 | 1.63E-12 | 0.13757 |
| Citrate cycle (TCA cycle) | 20 | 2 | 4.76E-11 | 10.322 | 9.53E-10 | 1.08E-10 | 0.13672 |
| Glycolysis / Gluconeogenesis | 26 | 4 | 1.96E-06 | 5.7074 | 2.94E-05 | 3.33E-06 | 0.12971 |
| Arginine biosynthesis | 14 | 2 | 7.98E-05 | 4.0978 | 0.000958 | 0.000109 | 0.11675 |
| Butanoate metabolism | 15 | 3 | 1.12E-21 | 20.95 | 3.37E-20 | 7.63E-21 | 0.11111 |
| Glutathione metabolism | 28 | 2 | 4.25E-08 | 7.3717 | 8.07E-07 | 8.50E-08 | 0.10839 |
| Arginine and proline metabolism | 38 | 3 | 6.50E-05 | 4.1874 | 0.000844 | 0.0001 | 0.09812 |
| Glycerophospholipid metabolism | 36 | 2 | 1.02E-11 | 10.993 | 2.14E-10 | 2.47E-11 | 0.03519 |
| Primary bile acid biosynthesis | 46 | 1 | 5.19E-05 | 4.2844 | 0.000727 | 8.41E-05 | 0.00758 |
| Aminoacyl-tRNA biosynthesis | 48 | 11 | 3.07E-26 | 25.513 | 1.04E-24 | 1.04E-24 | 0 |
| beta-Alanine metabolism | 21 | 1 | 1.47E-17 | 16.832 | 3.98E-16 | 6.26E-17 | 0 |
| Ubiquinone and other terpenoid-quinone biosynthesis | 9 | 1 | 1.01E-16 | 15.997 | 2.52E-15 | 3.42E-16 | 0 |
| Selenocompound metabolism | 20 | 1 | 6.12E-15 | 14.213 | 1.47E-13 | 1.89E-14 | 0 |
| Porphyrin and chlorophyll metabolism | 30 | 2 | 4.25E-08 | 7.3717 | 8.07E-07 | 8.50E-08 | 0 |
| Valine, leucine and isoleucine degradation | 40 | 4 | 9.73E-08 | 7.012 | 1.65E-06 | 1.84E-07 | 0 |
| Nitrogen metabolism | 6 | 2 | 7.98E-05 | 4.0978 | 0.000958 | 0.000109 | 0 |
| Pantothenate and CoA biosynthesis | 19 | 1 | 0.000217 | 3.6642 | 0.00195 | 0.000283 | 0 |
| Valine, leucine and isoleucine biosynthesis | 8 | 3 | 0.010619 | 1.9739 | 0.08495 | 0.013372 | 0 |
| Cysteine and methionine metabolism | 33 | 1 | 0.02067 | 1.6847 | 0.14469 | 0.0251 | 0 |
| Galactose metabolism | 27 | 1 | 0.027258 | 1.5645 | 0.16355 | 0.030893 | 0 |

Table S 13. Statistical analysis of metal ions concentration in dependence of menopausal and health status.

| **Comparison** | **Menopause** | **N=** | Group | As | Ca | Cu | Fe | Mg | Se | Zn |
| --- | --- | --- | --- | --- | --- | --- | --- | --- | --- | --- |
| Pre vs Post | Combined | (Pre= 88,  Post= 170) | Pre | 0.003 | 92.650 | 1.170 | 1.070 | 20.250 | 0.291 | 0.917 |
|  |  |  | Post | 0.085 | 96.650 | 1.180 | 1.125 | 21.100 | 0.332 | 0.925 |
|  |  |  | p value | 3.59E-03^b^ | 1.73E-06^b^ | 9.47E-01^b^ | 1.85E-01^b^ | 2.03E-04^b^ | 4.11E-01^b^ | 6.52E-01^b^ |
|  |  |  | FDR | 8.37E-03 | 1.21E-05 | 9.47E-01 | 3.24E-01 | 7.09E-04 | 5.76E-01 | 7.61E-01 |
| HC vs BC | Pre-meno | (HC= 56,  BC= 32) | HC | 0.003 | 92.659 | 1.180 | 1.115 | 19.600 | 0.257 | 0.932 |
|  |  |  | BC | 0.003 | 94.934 | 1.155 | 0.986 | 20.950 | 0.364 | 0.913 |
|  |  |  | p value | 1.40E-01^b^ | 1.54E-01^a^ | 3.29E-01^b^ | 5.55E-01^b^ | 4.51E-02^b^ | 8.24E-02^b^ | 5.89E-01^a^ |
|  |  |  | FDR | 2.70E-01 | 2.70E-01 | 4.61E-01 | 5.89E-01 | 2.70E-01 | 2.70E-01 | 5.89E-01 |
| HC vs BC | Post-meno | (HC= 44,  BC= 126) | HC | 0.039 | 95.850 | 1.195 | 1.160 | 21.350 | 0.274 | 1.020 |
|  |  |  | BC | 0.098 | 97.100 | 1.170 | 1.105 | 21.100 | 0.391 | 0.903 |
|  |  |  | p value | 5.00E-01^b^ | 2.20E-01^b^ | 4.29E-01^b^ | 4.65E-01^b^ | 2.23E-01^b^ | 8.12E-03^b^ | 1.02E-04^b^ |
|  |  |  | FDR | 5.00E-01 | 3.90E-01 | 5.00E-01 | 5.00E-01 | 3.90E-01 | 2.84E-02 | 7.15E-04 |

^a^Parametric test, ^b^Non-parametric.


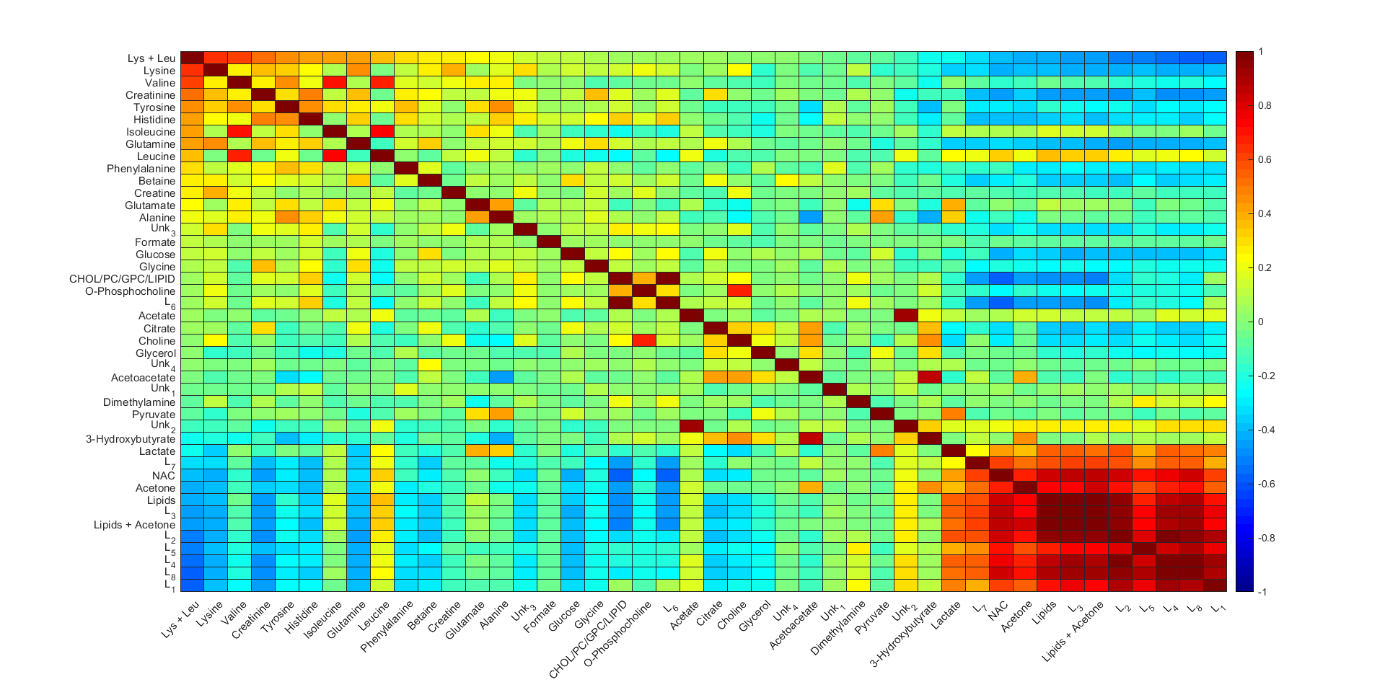


Figure S 1**.** Correlation between resonance signals for studied samples without distinction for groups.

**
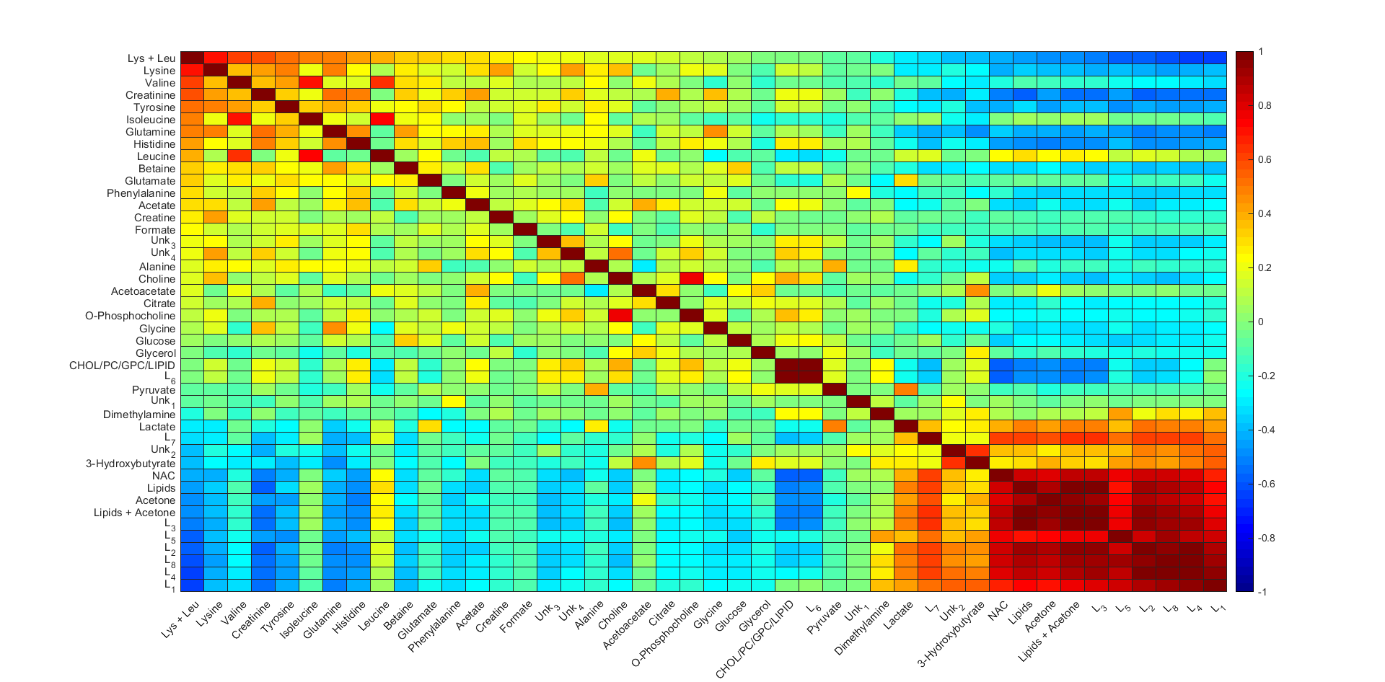
**

Figure S 2**.** Correlation between resonance signals for studied control group samples.

**
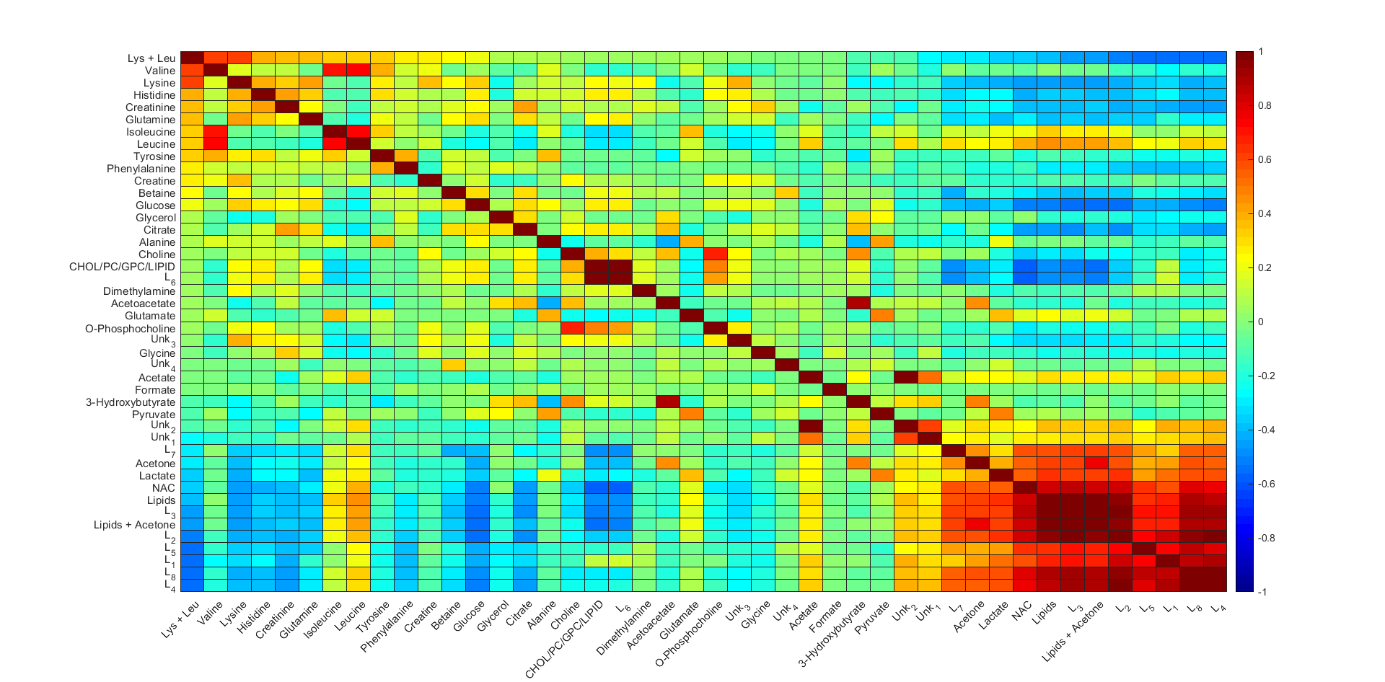
**

Figure S 3. Correlation between resonance signals for studied breast cancer groups samples.


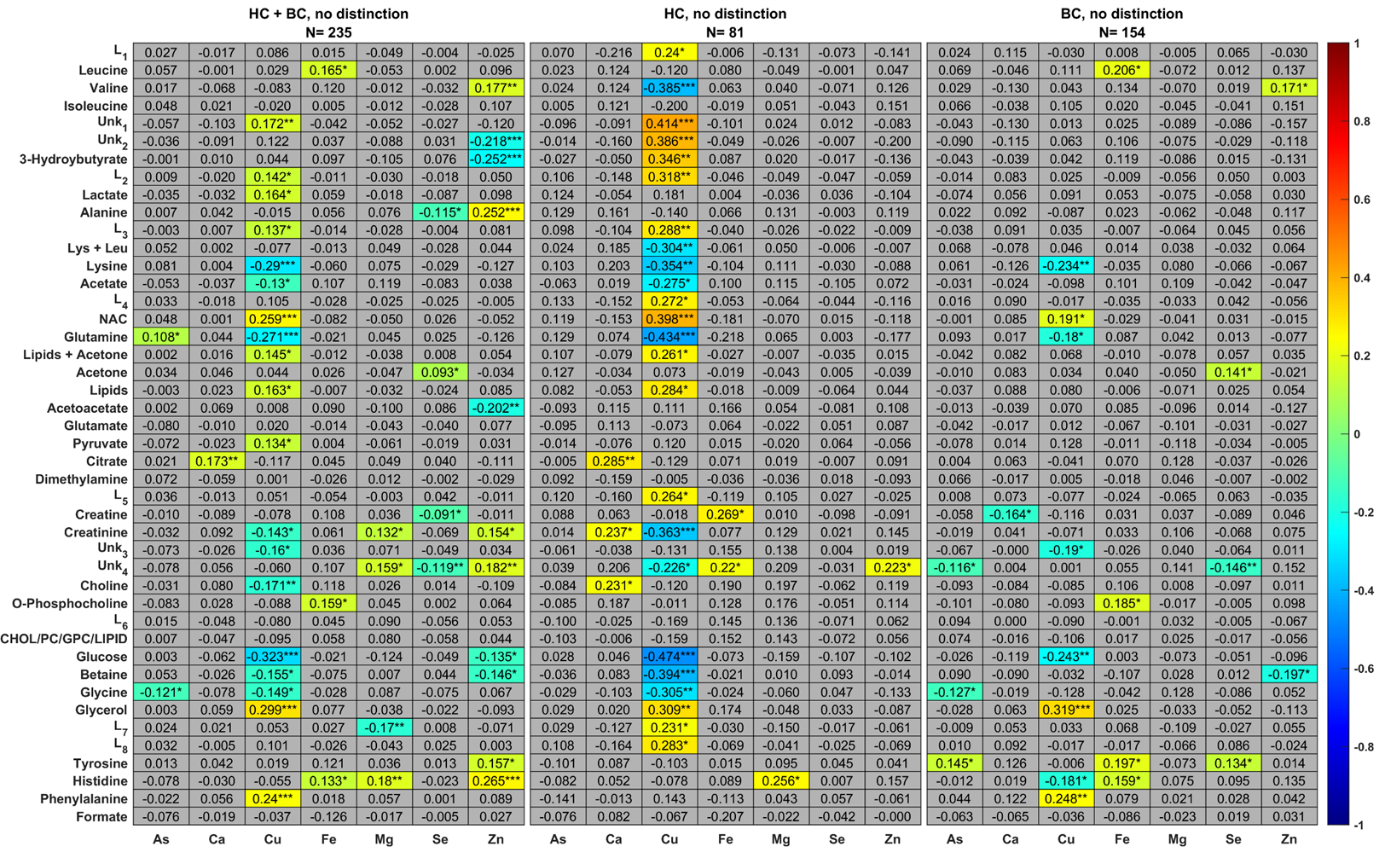


Figure S 4. The correlation between metal ions concertation and metabolites relative integral in studied patients groups without distinction of menopausal status. Grey– not statistically significant, color - statistically significant p value *< 0.05, **<0.01, ***<0.001. Color gradient corresponds to correlation coefficient.


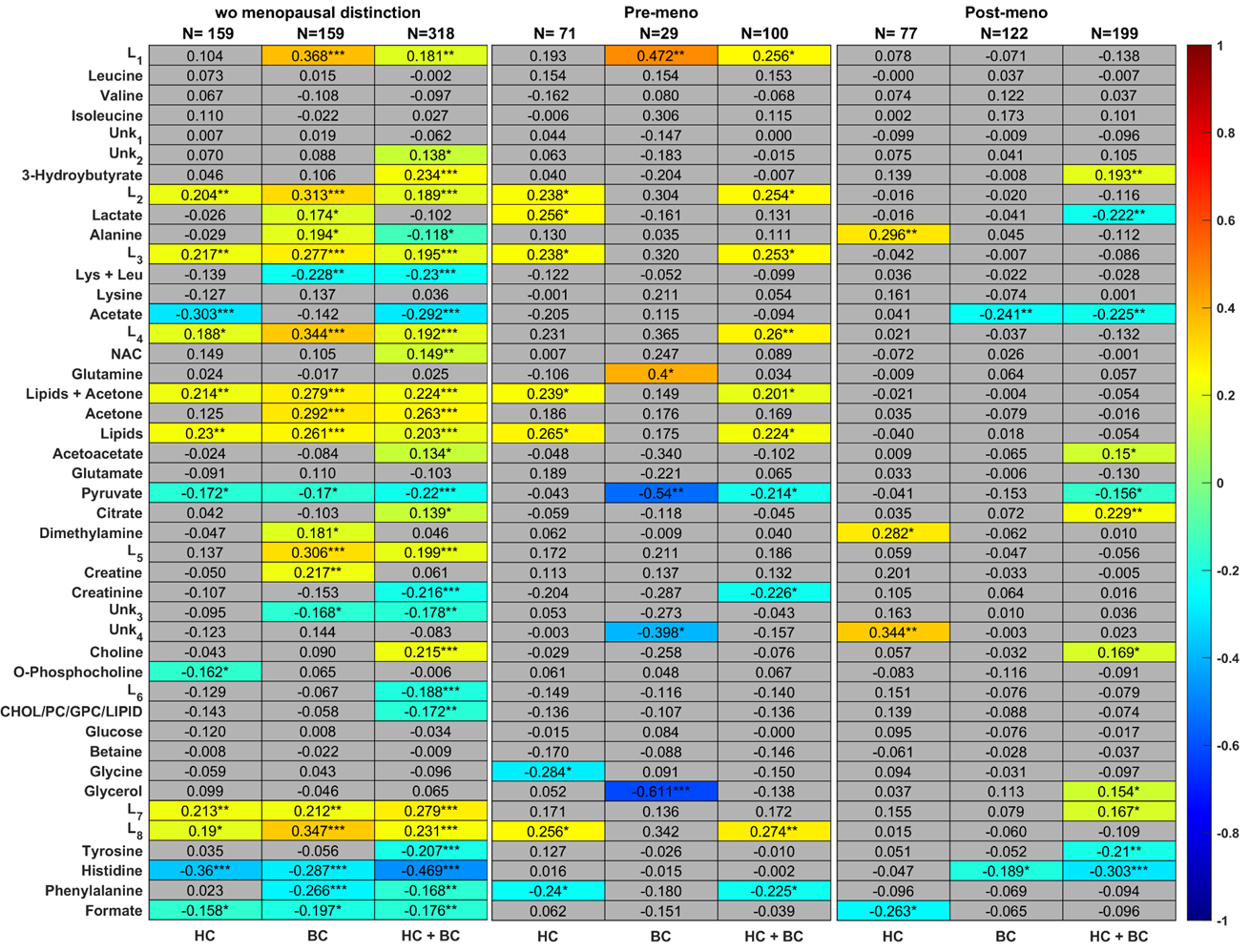


Figure S 5. The spearman correlation coefficients heatmap for age and metabolites relative integral for specific groups and conditions. Grey– not statistically significant, color - statistically significant p value *< 0.05, **<0.01, ***<0.001. Color gradient corresponds to correlation coefficient.


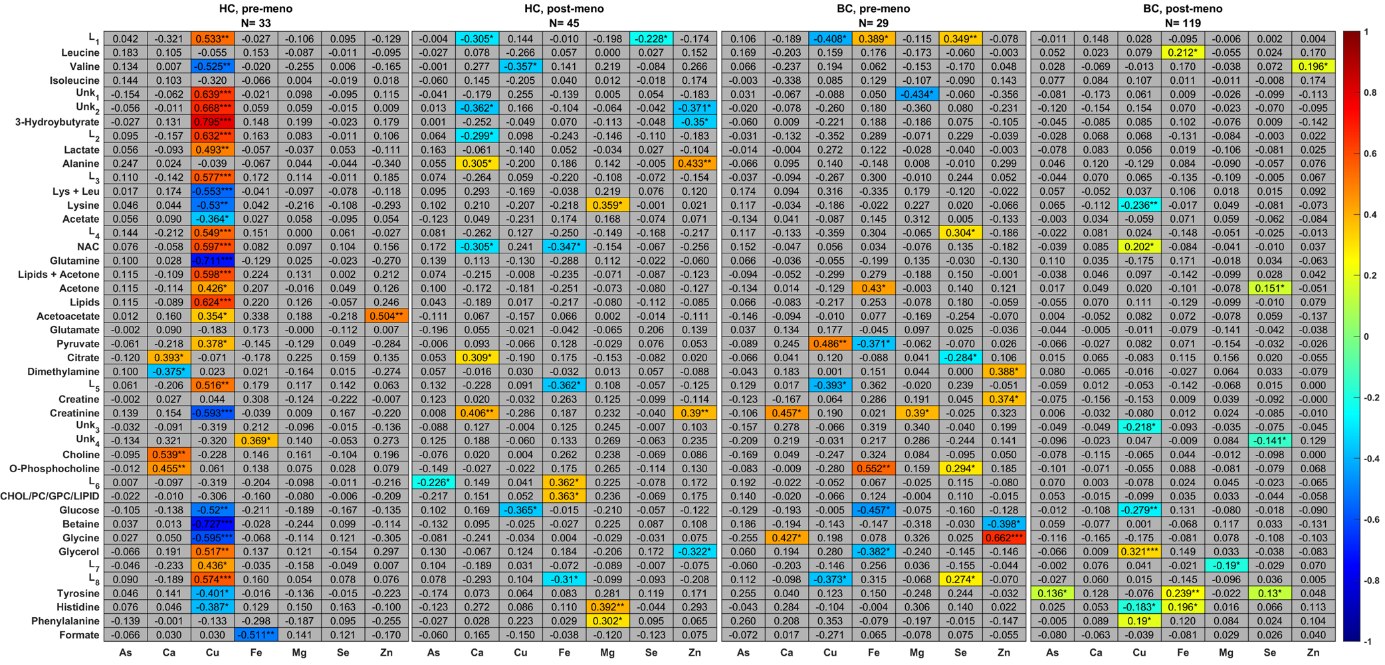


Figure S 6. The correlation between metal ions concertation and metabolites relative integral in control and breast cancer observations in dependence of menopausal status. Grey– not statistically significant, color - statistically significant p value *< 0.05, **<0.01, ***<0.001. Color gradient corresponds to correlation coefficient.
